# Supplementary material for: Circulating extracellular vesicle isomiR signatures predict therapy response in patients with multiple myeloma
Source: Cell Rep Med. 2025 Sep 16;6(10):102358. doi: 10.1016/j.xcrm.2025.102358 (PMC12629820; doi:10.1016/j.xcrm.2025.102358)
Supplement: Document S1. Figures S1–S6 and Tables S1 and S2 [file mmc1.pdf]

**Cell Reports Medicine, Volume *6***

## Supplemental information

# Circulating extracellular vesicle isomiR signatures predict therapy response in patients with multiple myeloma

**Cristina Gómez-Martín, Esther E.E. Drees, Monique A.J. van Eijndhoven, Nils J. Groenewegen, Steven Wang, Sandra A.W.M. Verkuijlen, Jan R.T. van Weering, Ernesto Aparicio-Puerta, Leontien Bosch, Kris A. Frerichs, Christie P.M. Verkleij, Marie J. Kersten, Josée M. Zijlstra, Daphne de Jong, Catharina G.M. Groothuis-Oudshoorn, Michael Hackenberg, Johan R. de Rooij, Niels W.C.J. van de Donk, and D. Michiel Pegtel**

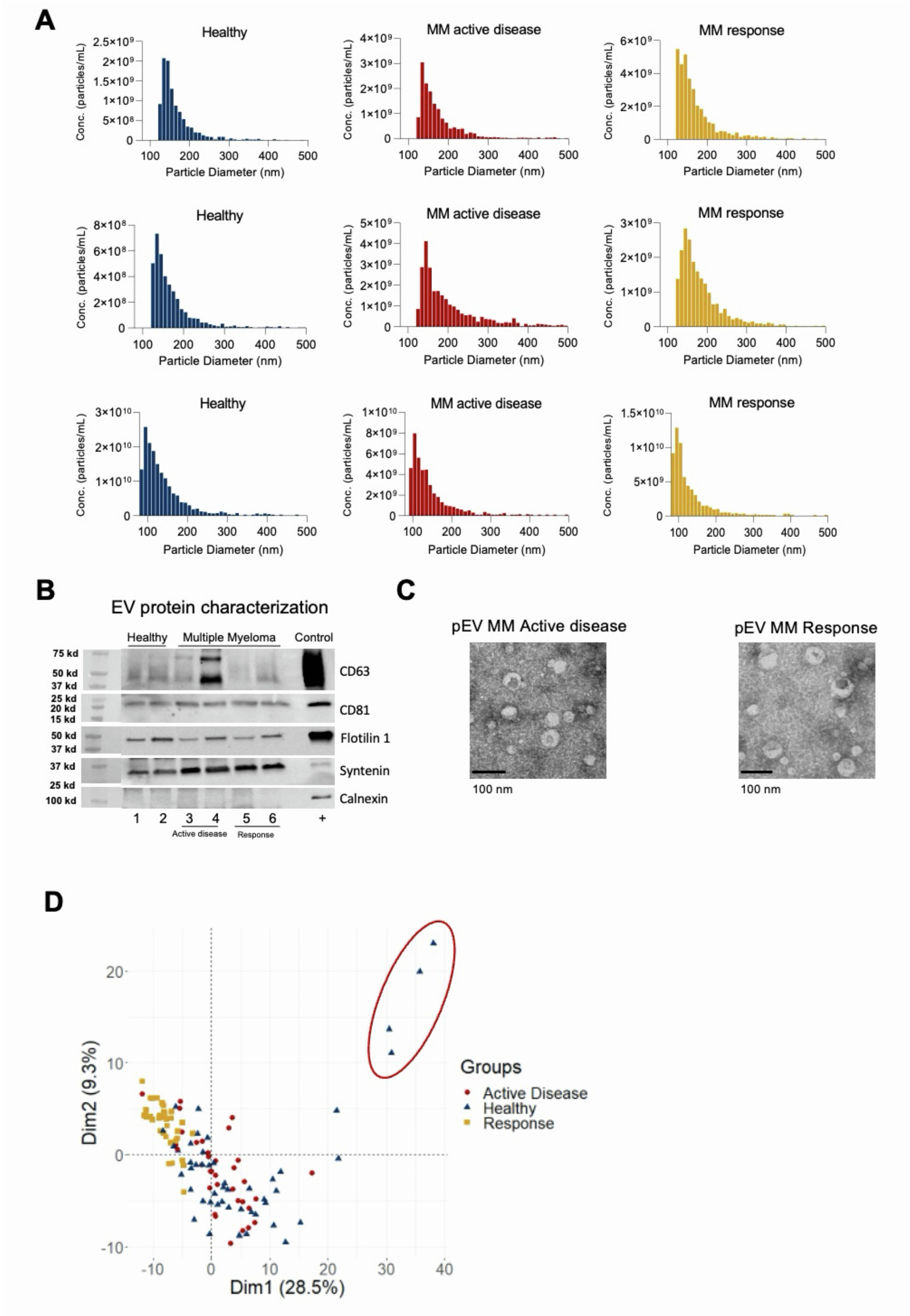


**Figure S1. EV characterization. Related to Figure 1 and STAR methods. A)** Particle size distribution of plasma EVs from healthy donors (left panels), MM patients with active disease (middle panels), and MM patients who achieved response to therapy (right panels), using Exoid. **B)** Western blot analysis of plasma EVs from healthy donors and MM patients with active disease or with clinical response. A B-cell lysate serves as a positive control. **C)** TEM analysis of pEVs from patients with MM active disease (left) or clinical response (right). Scale bar indicates 100 nm. **D)** PCA of all included samples, showing 4 outliers (red circle) belonging to healthy group.

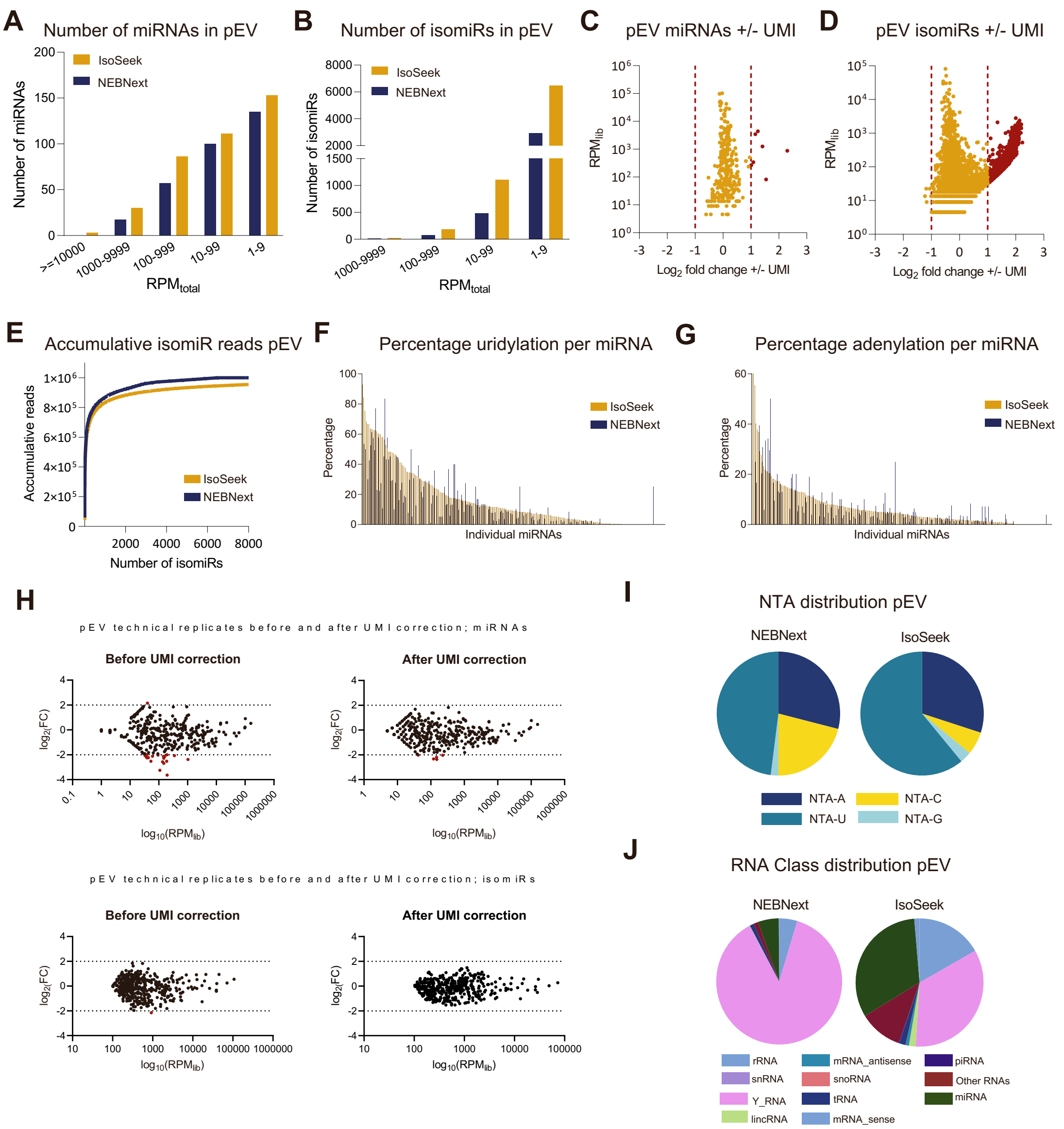


**Figure S2. IsoSeek improves the detection of miRNA and variants (isomiRs) at single nucleotide resolution in patient plasma EVs. Related to Figure 1 and STAR methods. A)** Number of different miRNAs detected in pEVs libraries prepared with NEBNext (blue) or IsoSeek (yellow), sorted by abundance ($RPM_{total}$). Data shown is the average of n=2 for each procedure. **B)** Same as in (A) but for isomiRs as separate features. **C)** Differential expression analysis of pEVs-miRNAs using 5N-adapters with or without UMI correction. Each dot represents a miRNA. Representative data is shown. **D)** Same as in (C) but for isomiRs as separate features. **E)** Accumulative normalized isomiR reads from pEVs libraries prepared using NEBNext (blue) and IsoSeek (yellow). The results shown are the average of n=2 for both procedures. **F)** Percentage of uridylation for each miRNA in pEVs libraries prepared using NEBNext (blue) or IsoSeek (yellow). The NTA-U reads were divided by the total normalized reads for each miRNA. Each line represents a miRNA, sorted by abundance based on IsoSeek. Data shown is the average of n=2 for each library preparation procedure. Analysis includes miRNAs >= 10 RPM (total reads) in all samples. **G)** Same as in (F) but for the percentage of adenylation. **H)** Differential expression analysis of pEVs-miRNAs (top) or pEVs-isomiRs (bottom) of technical replicates using 5N-adapters with UMI (blue) or without UMI correction (red). Each line represents a miRNA, sorted by abundance based on the DEA with UMI correction. Representative data is shown. Analysis only includes miRNAs detected in both samples, with a cut-off for miRNAs (top) of 1RPM and a cut-off for isomiRs (bottom) of 100 RPM. **I)** NTA distribution in pEVs libraries prepared with NEBNext (left) and IsoSeek (right). Data shown is the average of n=2 for each procedure. **J)** Small RNA species distribution in pEVs libraries prepared with NEBNext (left) and IsoSeek (right). Data shown is the average of n=2 for each procedure.

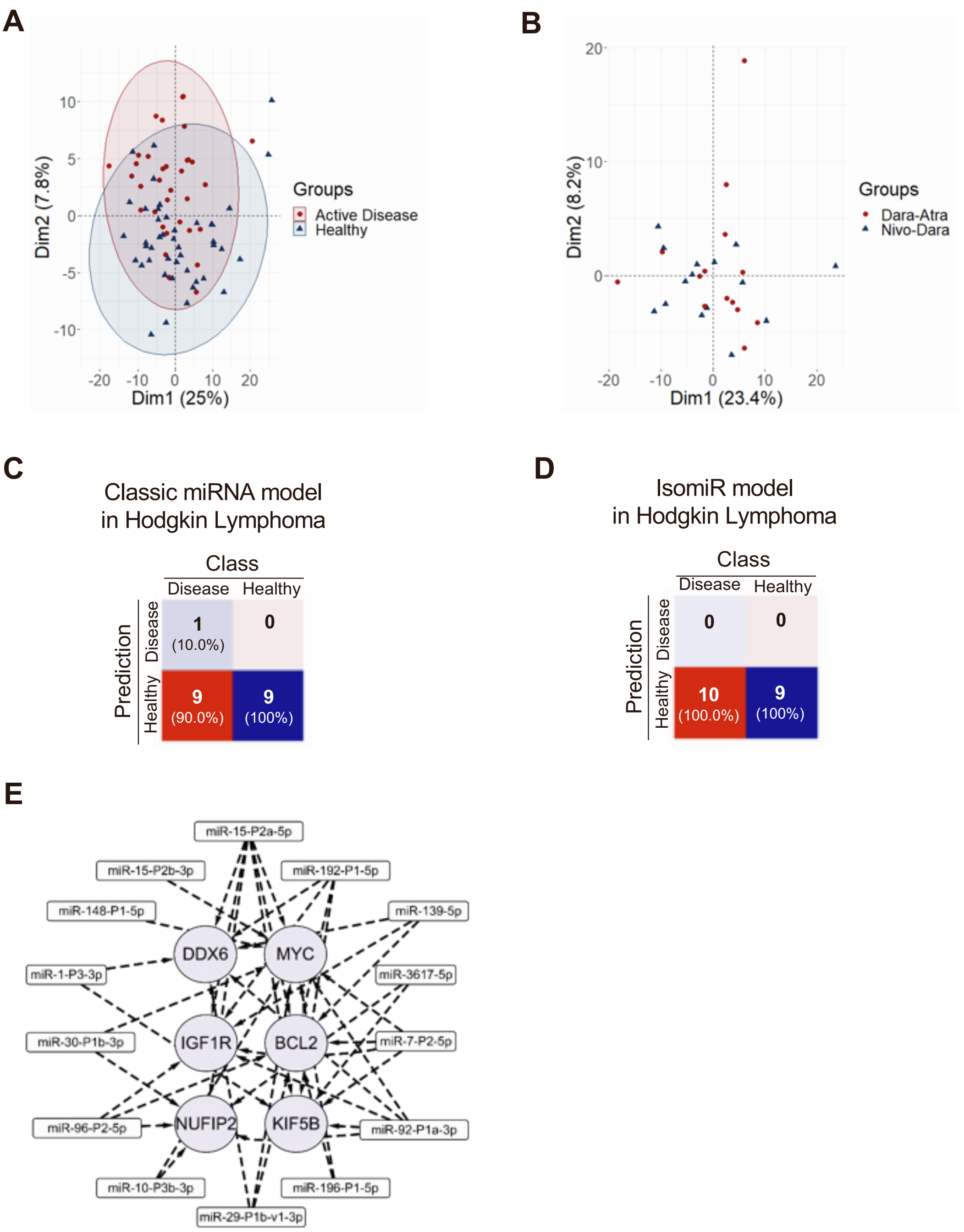


**Figure S3. Related to Figure 2. A)** Principal component analysis with all samples from MM patients with active disease and from healthy donors. **B)** Principal component analysis with all samples from MM patients with active disease annotated by treatment **C** and **D)** Performance of the classic miRNA MM disease model **(C)** and isomiR model **(D)** on Hodgkin Lymphoma active disease samples as well as a set of healthy controls. **E)** Full miRNA-gene-target network of the classic miRNA disease detection model.

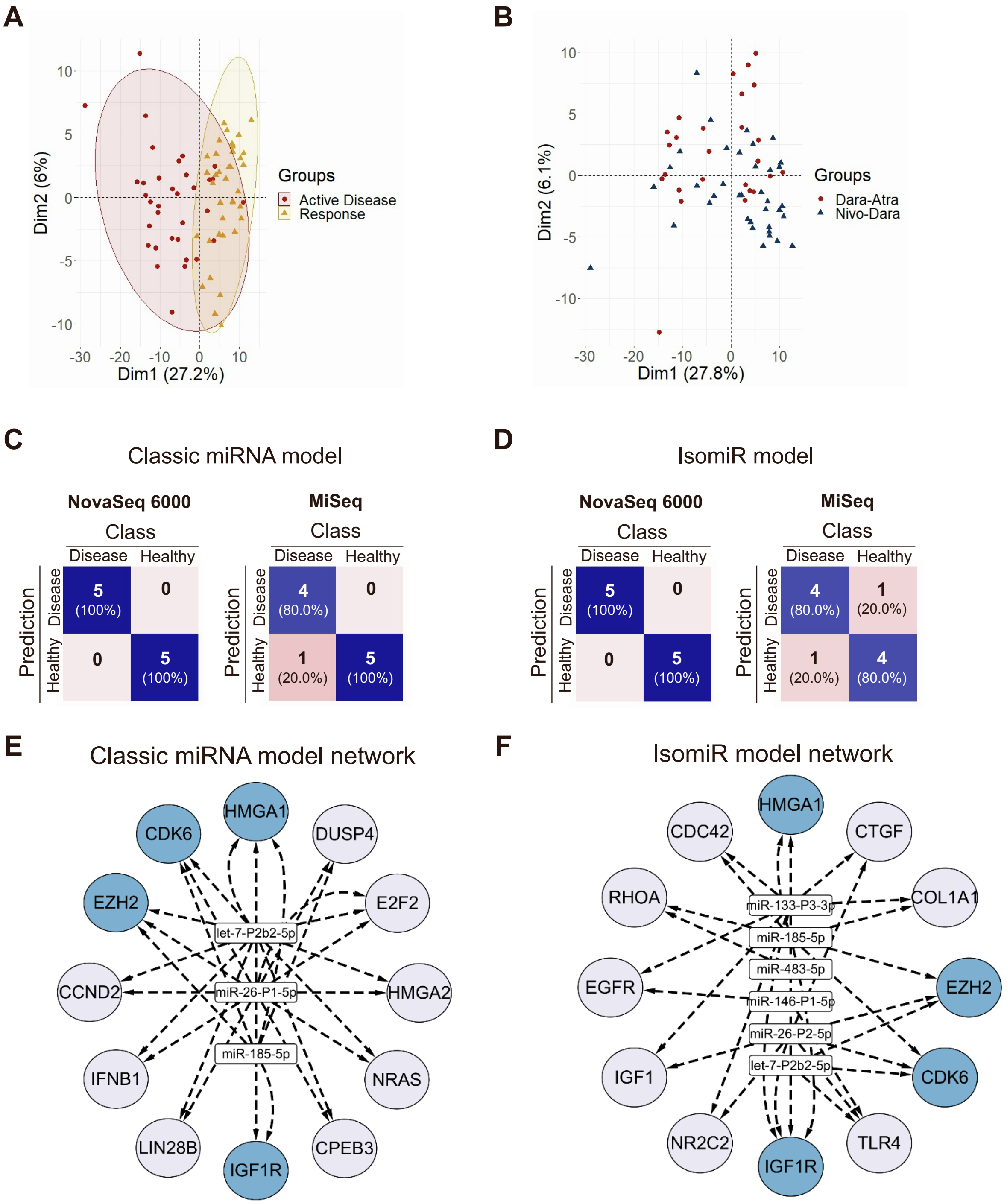


**Figure S4. Related to Figure 3. A)** Principal component analysis with all samples from MM patients with active disease and with clinical response. **B)** Principal component analysis with all MM samples (AD and response) annotated by treatment. **C-D)** Performance of the classic miRNA MM disease model **(C)** and isomiR model **(D)** on resequenced samples using the MiSeq sequencing platform. **E)** Full miRNA-gene-target network of the classic miRNA disease detection model. **F)** Full miRNA-gene-target network of the IsomiR miRNA disease detection model.

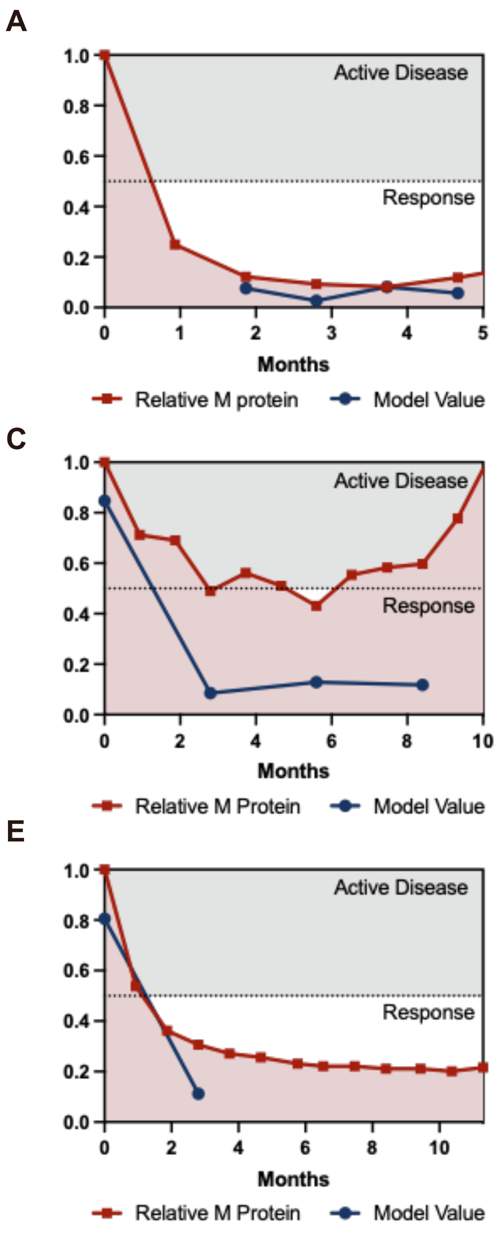


**Figure S5. Related to Figure 4. A-E)** pEV-isomiR model predictions over time compared to M-protein levels for four individual patients, showing that the model closely tracks the M-protein metric, demonstrating its robustness.

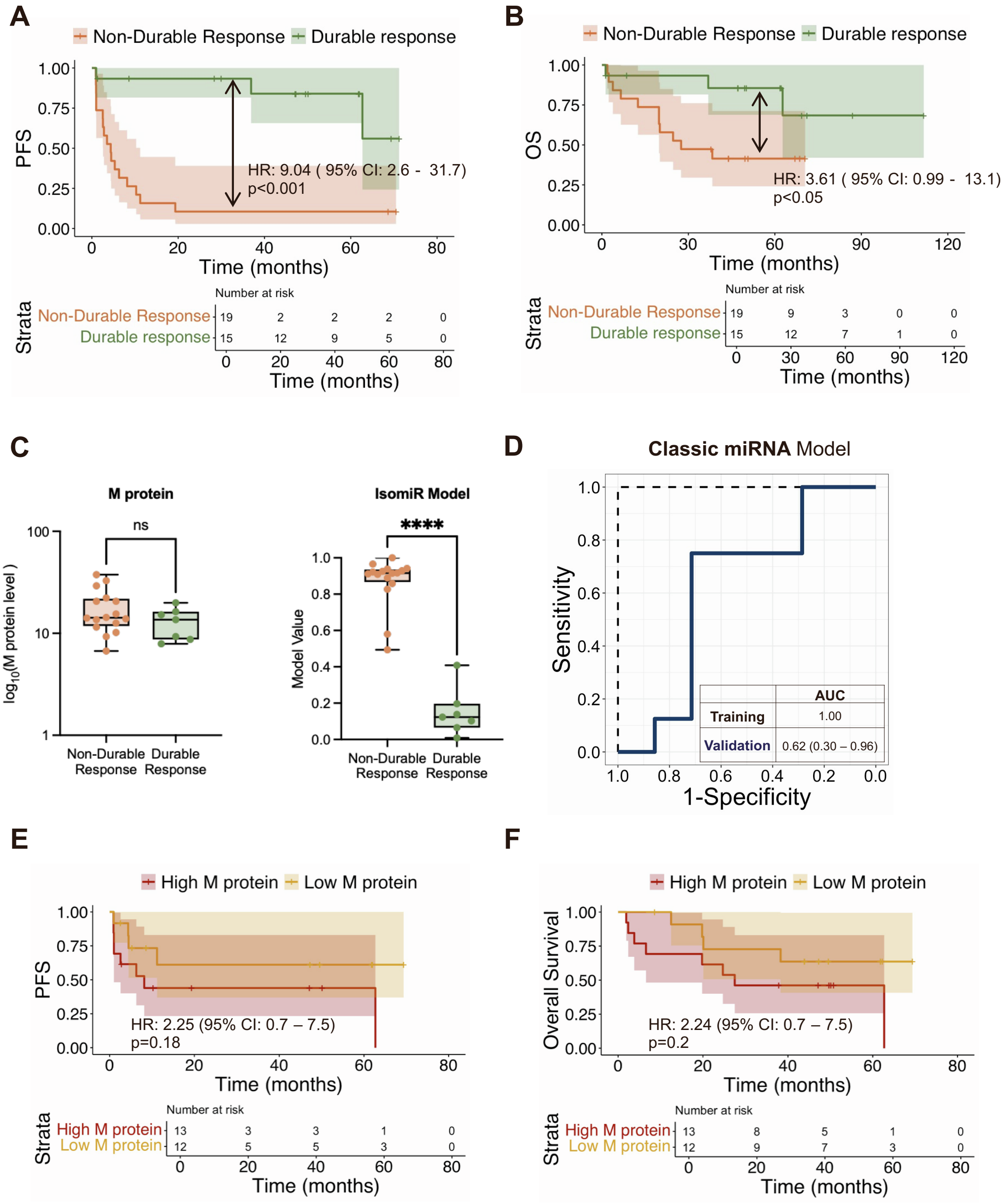


**Figure S6. Related to Figure 5. A)** Progression-free survival (PFS) of the total set of patients included in the model in Figure 6, including both RRMM and NDMM patients (n=34). The survival curve was computed using the Kaplan-Meier method. The hazard ratio (HR) calculated by Cox-regression is 9.04 (CI:2.6-31.7, p<0.001). **B)** Overall survival (OS) analysis of the total set of patients included in the model in Figure 6, including both RRMM and NDMM patients (n=34). The survival curve was computed using the Kaplan-Meier method. The hazard ratio (HR) calculated by Cox-regression is 3.61 (CI:0.99-13.1, p<0.05). **C)** (Left) Log10-transformed baseline M-protein levels in Non-Durable Response (orange) and Durable Response (green) patients. No significant difference was observed (t-test, n.s.) between the two groups. (Right) IsomiR prediction model value in Non-Durable response (orange) and Durable Response (green). Significant difference was observed (t-test, p-value < 0,0001) between the two groups. **D)** ROC curve of the classic miRNA durable response prediction model. The training model (dotted line) had and AUC of 1.0, but could not be validated in the independent validation set (AUC: 0.62). **E)** Progression-free survival (PFS) of RRMM patients (n=25) divided by M-protein values (high or low). The survival curve was computed using the Kaplan-Meier method. The hazard ratio (HR) calculated by Cox-regression is 2.25 (CI:0.7-7.5, p=0.18). **F)** Overall survival (OS) analysis of RRMM patients (n=25). The survival curve was computed using the Kaplan-Meier method. The hazard ratio (HR) calculated by Cox-regression is 2.24 (CI:0.7-7.5, p=0.2).

| **Patients with MM included in disease detection model (Figure 2)** | **Training cohort** | **Validation Cohort** |
| --- | --- | --- |
| **Number of Samples** | ***22 patients 22 samples*** | ***16 patients 18 samples*** |
| **Gender** | 9 male<br>13 female | 11 male<br>5 female |
| **Age** | Median: 62 yrs<br>Range: 47-80 yrs | Median: 66 yrs<br>Range: 54-80 yrs |
| **Treatment line** | Median: 5<br>Range: 0-11 | Median: 4<br>Range: 0-9 |
| **Treatment regimen (treatment at time of collection samples)** | | |
| **RRMM Daratumumab trial patients** | | |
| NIVO-DARA +/- low-dose cyclophosphamide | 8 | 7 |
| DARA-ATRA | 10 | 8 |
| **Biobank (Biolymph VUmc )** | | |
| ***NDMM*** | | |
| VTd followed by HDM/ASCT | 2 | 0 |
| VCd followed by HDM/ASCT | 1 | 1 |
| ***RRMM*** | | |
| Daratumumab (+/- lenalidomide/dexamethasone) | 1 | 0 |
| **Monoclonal protein** | | |
| IgG kappa | 14 | 11 |
| IgG lambda | 3 | 1 |
| IgA kappa | 1 | 0 |
| IgA lambda, non/oligo-secretor | 1 | 1 |
| FLC kappa | 3 | 2 |
| FLC lambda | 0 | 1 |
| **High-risk cytogenetic abnormality** | | |
| High risk | 12 | 7 |
| Standard risk | 4 | 3 |
| Unknown | 6 | 6 |

| **Healthy donors included in disease detection model (Figure 2)** | **Training cohort** | **Validation Cohort** |
| --- | --- | --- |
| **Gender** | 14 male<br>15 female | 9 male<br>6 female |
| **Age** | Median: 63 yrs<br>Range: 45 – 78 yrs | Median: 61 yrs<br>Range: 48 – 79 yrs |

**Table S1: Response status of the training and validation set for disease detection model in Figure 2.** **Abbreviations:** HDM = high-dose melphalan; ASCT = autologous stem cell transplantation; NIVO = nivolumab; DARA = daratumumab; ATRA = all trans retinoic acid; VTd = Bortezomib-thalidomide-dexamethasone; VCd = Bortezomib-cyclophosphamide-dexamethasone. Based on the criteria proposed by Sonneveld et al. Blood 2016. High risk cytogenetics is defined by the presence of t(4;14), t(14;16), t(14;20), del(17/17p), and/or gain(1q).

| Patients with MM included in response assessment model (Figure 3) | Training set RRMM | | Validation RRMM | | Prospective validation RRMM and NDMM | |
|---|---|---|---|---|---|---|
| | *Active Disease* | *Response* | *Active Disease* | *Response* | *Active Disease* | *Response* |
| | ***20 patients 20 samples*** | ***11 patients 22 samples*** | ***13 patients 15 samples*** | ***7 patients 13 samples*** | ***6 patient 6 samples*** | ***11 patients 11 samples*** |
| **Gender** | 9 male 11 female | 3 male 8 female | 8 male 5 female | 3 male 4 female | 3 male 3 female | 7 male 4 female |
| **Age** | Median: 67 yrs Range: 47-80 yrs | Median: 60 yrs Range: 55-77 yrs | Median: 66 yrs Range: 54-80 yrs | Median: 62 yrs Range: 54-74 yrs | Median: 59 yrs Range: 57-87 yrs | Median: 63 yrs Range: 34-80 yrs |
| **Treatment line** | Median: 4 Range: 2-11 | Median: 5 Range: 3-8 | Median: 4 Range: 3-9 | Median: 4 Range: 3-8 | Median: 0 Range: 0-7 | Median: 1 Range: 1-10 |
| **Treatment regimen (treatment at time of sample collection)** | | | | | | |
| **RRMM Daratumumab trial patients** | | | | | | |
| NIVO-DARA +/- low-dose cyclophosphamide | 10 | 7 | 5 | 6 | | |
| DARA-ATRA | 10 | 4 | 8 | 1 | | |
| **Biobank (Biolymph VUmc / AMC)** | | | | | | |
| ***<u>NDMM</u>*** | | | | | | |
| VTd followed by HDM/ASCT | | | | | 2 | 0 |
| VCd followed by HDM/ASCT | | | | | 2 | 0 |
| Lenalidomide maintenance after HDM/ASCT | | | | | 0 | 2 |
| KTd followed by HDM/ASCT (CarthaDex trial) | | | | | 0 | 0 |
| KRd followed by HDM/ASCT (HOVON503 trial, arm B) | | | | | 0 | 1 |
| ***<u>RRMM</u>*** | | | | | | |
| Daratumumab (+/-lenalidomide/dexamethasone) | | | | | 2 | 2 |
| Standard of care CARTITUDE-4 trial regimen | | | | | 0 | 1 |
| TRIMM-2 trial | | | | | 0 | 1 |
| VCd followed by DLI | | | | | 0 | 1 |
| PCd | | | | | 0 | 1 |
| EPd | | | | | 0 | 1 |
| DVd | | | | | 0 | 1 |
| **Monoclonal protein** | | | | | | |
| IgG kappa | 13 | 7 | 9 | 4 | 5 | 3 |
| IgG lambda | 4 | 2 | 0 | 1 | 0 | 0 |

| | | | | | | |
|---|---|---|---|---|---|---|
| IgA kappa | 1 | 0 | 0 | 0 | 0 | 1 |
| IgA lambda, non/oligo-secretor | 0 | 0 | 1 | 0 | 0 | 2 |
| FLC kappa | 1 | 2 | 3 | 2 | 1 | 5 |
| FLC lambda | 1 | 0 | 0 | 0 | 0 | 0 |
| **High-risk cytogenetic abnormality** | | | | | | |
| High risk | 12 | 6 | 7 | 4 | 0 | 2 |
| Standard risk | 2 | 2 | 3 | 1 | 2 | 1 |
| Unknown | 7 | 3 | 3 | 2 | 4 | 8 |

| | | | | | | |
|---|---|---|---|---|---|---|
| **Distribution of the samples per analysis group** | | | | | | |
| **Clinical trial samples** | | | | | | |
| **Active Disease (AD) Group** | **20** | | **15** | | | |
| NIVO-DARA (ND) baseline | 10 | | 6 | | | |
| DARA-ATRA (DA) baseline | 6 | | 8 | | | |
| DARA-ATRA (DA) post ATRA | 4 | | 1 | | | |
| **Response group** | | **22** | | **13** | | |
| Partial response | | 10 | | 7 | | |
| Very good partial response | | 8 | | 4 | | |
| Complete response | | 4 | | 2 | | |
| ***Biobank Samples*** | | | | | | |
| **Active Disease (AD) Group** | | | | | **6** | |
| Newly Diagnosed | | | | | 4 | |
| Progressive disease | | | | | 2 | |
| **Response group** | | | | | | **11** |
| Stable disease | | | | | | 1 |
| Partial response | | | | | | 2 |
| Very good partial response | | | | | | 3 |
| Complete response | | | | | | 5 |

**Table S2: Response status of the training, validation and prospective validation set for the response assessment model from Figure 3.**
**Abbreviations:** HDM = high-dose melphalan; ASCT = autologous stem cell transplantation; NIVO = nivolumab; DARA = daratumumab; ATRA = all trans retinoic acid; VTd = Bortezomib-thalidomide-dexamethasone; VCd = Bortezomib-cyclophosphamide-dexamethasone; KTd = Carfilzomib-thalidomide-dexamethasone; KRd = Carfilzomib-lenalidomide-dexamethasone ; Standard of care CARTITUDE-4, Arm = either bortezomib or daratumumab, with pomalidomide and dexamethasone; TRIMM-2 trial = Talquetamab– daratumumab–pomalidomide; DLI = donor lymphocyte infusion; PCd = Pomalidomide-cyclophosphamide-dexamethasone, EPd = Elotuzumab-pomalidomide-dexamethasone; DVd = Daratumumab-bortezomib-dexamethasone. High risk cytogenetics is defined by the presence of t(4;14), t(14;16), t(14;20), del(17/17p), and/or gain(1q). Second part of the table depicts the sample distribution between the different cohorts including the response status of the sample at time of sample inclusion in the model.
