# Supplementary material for: Circulating extracellular vesicle isomiR signatures predict therapy response in patients with multiple myeloma
Source: Cell Rep Med. 2025 Sep 16;6(10):102358. doi: 10.1016/j.xcrm.2025.102358 (PMC12629820; doi:10.1016/j.xcrm.2025.102358)
Supplement: Document S2. Article plus supplemental information [file mmc2.pdf]

Cell Reports Medicine

Article

Circulating extracellular vesicle isomiR signatures predict therapy response in patients with multiple myeloma

Graphical abstract

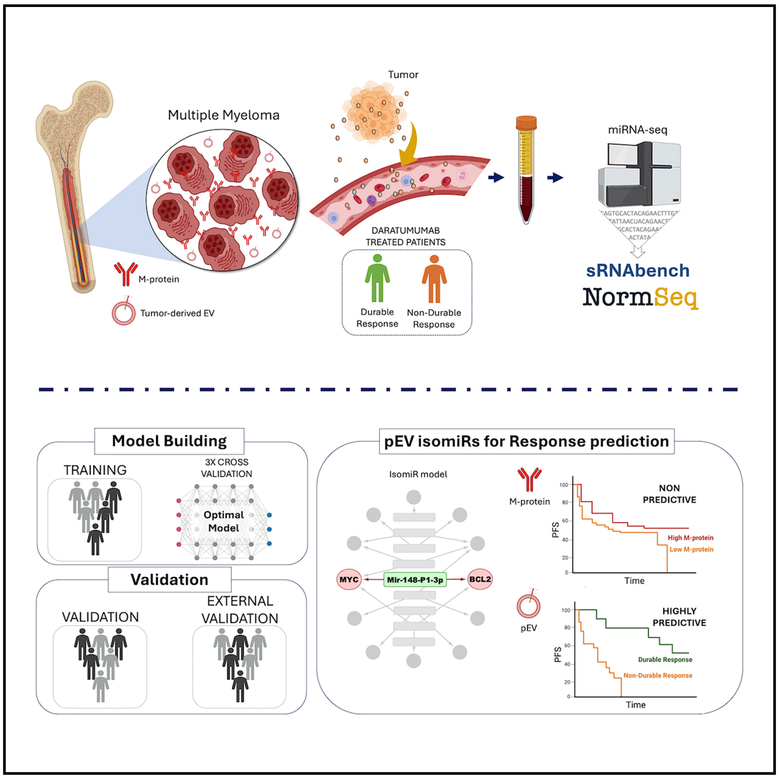

Highlights

- IsoSeek detects thousands of isomiRs in plasma EVs from MM patients
- EVs from MM patients are enriched in plasma B cell-derived transcripts
- A machine learning strategy builds biologically driven EV isomiR-gene target networks
- EV-IsomiR signature predicts durable response to daratumumab in real-world MM patients

Authors

Cristina Gómez-Martín, Esther E.E. Drees, Monique A.J. van Eijndhoven, ..., Johan R. de Rooij, Niels W.C. J. van de Donk, D. Michiel Pegtel

Correspondence

d.pegtel@amsterdamumc.nl

In brief

Current diagnostic methods fail to predict which patients with multiple myeloma will benefit from daratumumab-containing treatment regimens. Gómez-Martín et al. demonstrate that a machine learning strategy applied to small RNA sequencing data from plasma extracellular vesicle fractions can generate biologically motivated isomiR signatures that predict treatment response.

Check for updates

Gómez-Martín et al., 2025, Cell Reports Medicine 6, 102358
October 21, 2025 © 2025 The Author(s). Published by Elsevier Inc.
https://doi.org/10.1016/j.xcrm.2025.102358

CellPress

Cell Reports Medicine

CellPress
OPEN ACCESS

Article

Circulating extracellular vesicle isomiR signatures predict therapy response in patients with multiple myeloma

Cristina Gómez-Martín,[1,2,11] Esther E.E. Drees,[1,2,11] Monique A.J. van Eijndhoven,[1,2] Nils J. Groenewegen,[1,2,3] Steven Wang,[1,2] Sandra A.W.M. Verkuijlen,[1,2] Jan R.T. van Weering,[4] Ernesto Aparicio-Puerta,[5,6,7,8] Leontien Bosch,[1,2] Kris A. Frerichs,[2,9] Christie P.M. Verkleij,[2,9] Marie J. Kersten,[2,9] Josée M. Zijlstra,[2,9] Daphne de Jong,[1,2] Catharina G.M. Groothuis-Oudshoorn,[10] Michael Hackenberg,[3,5,6,7,8] Johan R. de Rooij,[3] Niels W.C.J. van de Donk,[2,9] and D. Michiel Pegtel[1,2,3,12,*]

[1]Amsterdam UMC Location Vrije Universiteit Amsterdam, Department of Pathology, Boelelaan 1117, Amsterdam, the Netherlands
[2]Cancer Center Amsterdam, Program Imaging and Biomarkers, Amsterdam, the Netherlands
[3]ExBiome B.V., Amsterdam, the Netherlands
[4]Amsterdam UMC Location Vrije Universiteit Amsterdam, Department of Human Genetics, Amsterdam Neuroscience - Neurodegeneration, De Boelelaan 1085, 1081 HV Amsterdam, the Netherlands
[5]Computational Epigenomics and Bioinformatics, Department of Genetics, University of Granada, Granada, Spain
[6]Bioinformatics Laboratory, Biotechnology Institute, Centro de Investigación Biomédica, PTS, Avda. del Conocimiento s/n, 18100 Granada, Spain
[7]Instituto de Investigación Biosanitaria ibs. GRANADA, University of Granada, 18071 Granada, Spain
[8]Excellence Research Unit "Modelling Nature" (MNat), University of Granada, 18071 Granada, Spain
[9]Amsterdam UMC, Location Vrije Universiteit Amsterdam, Department of Hematology, Amsterdam, the Netherlands
[10]Department of Health Technology and Services Research, Technical Medical Centre, University of Twente, Enschede, the Netherlands
[11]These authors contributed equally
[12]Lead contact
*Correspondence: d.pegtel@amsterdamumc.nl
https://doi.org/10.1016/j.xcrm.2025.102358

SUMMARY

Multiple myeloma (MM) is a plasma cell neoplasm characterized by high inter- and intra-patient clonal heterogeneity, leading to high variability in therapeutic responses. Minimally invasive biomarkers that predict response may help personalize treatment decisions. IsoSeek, a single-nucleotide resolution small RNA sequencing method can profile thousands of microRNAs (miRNAs) and their variants (isomiRs) from patient plasma-purified extracellular vesicles (EVs). Machine learning-generated miRNA/isomiR classifiers accurately predict therapeutic response in relapsed/refractory MM (RRMM) patients receiving daratumumab-containing regimens, achieving an area-under-the-curve of 0.98 (95% confidence interval [CI]:0.94–1.00). A classifier signature with the plasma cell-selective miR-148-3p, predicts durable response (≥6 months), progression-free (hazard ratio [HR]: 33.09, 95% CI: 4.2–262, $p < 0.001$), and overall survival (HR: 3.81, 95% CI: 1.05–13.99, $p < 0.05$). Targetome analysis connects the prognostic classifier to established MM drug targets BCL2 and MYC suggesting biological relevance. Thus, EV-isomiR sequencing in MM patients offers a tumor-naïve alternative to an invasive bone-marrow biopsy for predicting treatment outcome.

INTRODUCTION

Multiple myeloma (MM) is characterized by the uncontrolled proliferation of clonal plasma cells in the bone marrow (BM). The advent of immunomodulatory drugs (e.g., lenalidomide and thalidomide), proteasome inhibitors (bortezomib), and more recently, monoclonal antibodies (daratumumab), has improved survival.[1] While first-line treatments yield good responses, most patients eventually relapse. Despite improved response rates with follow up treatment regimens, individual variability persists, with some patients being primary refractory to certain therapies or early relapsing.[1,2] The shift from an incurable to a chronic disease will largely depend on improving biomarker strategies that accurately monitor therapeutic response and ideally predict favorable outcomes for the individual patients.

The current gold standard for monitoring response to therapy includes measuring M-protein and free light chains in blood and/or urine,[1] along with BM examination to confirm complete response or detect minimal residual disease (MRD). Achieving MRD negativity in the BM is associated with superior progression-free survival (PFS) and overall

© 2025 The Author(s). Published by Elsevier Inc.
This is an open access article under the CC BY license (http://creativecommons.org/licenses/by/4.0/).

survival (OS) compared to MRD-positive results.[3] However, BM MRD evaluation with next generation flow or next generation sequencing (NGS), is invasive and burdensome, limiting its sequential use, and may produce false-negative results due to multifocal disease, presence of extramedullary disease and/or poor quality of BM aspirates and biopsies.[3] Notably, sustained MRD negativity measured by NGS over time has shown superior prognostic value over a single time point MRD-negative result.[4] Therefore, there is an urgent need for easy interpretable, minimally invasive biomarkers that better predict which individual patient is responding or likely to respond to therapy.[5] Minimally invasive methods, including blood-based (targeted) mass spectrometry,[6] circulating tumor DNA (ctDNA),[7] circulating plasma cells[8] and cell-free microRNAs (miRNAs)[9] are under investigation for prognostic and predictive potential, as dynamic M-protein or free light chain measurements fall short in predicting outcome.

Beyond driver mutations, non-coding RNAs, particularly miRNAs, play a significant role in the transformation and drug resistance of malignant plasma cells.[10,11] miRNAs are promising cancer biomarker targets for liquid biopsy methods, as they are released by both living and dying malignant and stromal cells. A dynamic mixture of these cell-free miRNAs is stabilized in, and can be extracted from extracellular vesicles (EVs), secreted by metabolically active tumor cells.[12] The discovery of circulating miRNAs as cancer biomarkers involves sequencing either tumor-tissues biopsies or plasma, followed by quantitative reverse-transcription PCR (RT-qPCR) for detection.[11,13] Since individual miRNAs often lack tissue and cell-specificity, profiling methods are used to build multi-miRNA signatures, enhancing the accuracy for both disease detection[14] and response to therapy prediction.[15]

Measuring miRNAs bound to plasma EVs (pEVs) helps to reduce biological noise[16] and has shown prognostic value in newly diagnosed patients with MM.[9] miRNA profiling is typically performed using standard sequencing protocols, which are prone to ligation and amplification bias during library preparation.[17–19] Unfortunately, the lack of single-nucleotide resolution of standard sequencing and RT-qPCR precludes accurate identification and quantification of functional miRNA variants (isomiRs) and gene-targets.[20] In this study, we profiled pEV-bound isomiRs (pEV isomiRs) at single-nucleotide resolution using an in-house developed small RNA sequencing protocol, "IsoSeek," optimized for low-input liquid biopsy sources.[17,19] Upon extensive normalization and correction procedures,[21] we generated and validated pEV-isomiR signatures using machine learning to detect active MM, monitor treatment response, and predict durable response. Our method requires only 1 mL of blood plasma, is scalable to a standard diagnostic MiSeq Illumina platform, and offers some flexibility in pre-analytical conditions thereby simplifying implementation in clinical practice.

RESULTS

Immune-cell-derived small EVs are present in the circulation of patients with MM

pEVs may hold hundreds of miRNAs, yet non-standardized isolation and detection methods are prone to technical biases, hampering robust detection, validation, and clinical implementation.[13,22] To overcome these challenges, we developed and validated a standardized workflow to isolate pEVs from both patients with MM and healthy controls (Figure 1A; Table 1).

The EV-RNA analysis workflow (Figure 1B) integrates automated size-exclusion chromatography (SEC) for EV isolation with an optimized EV-RNA extraction protocol[12,13,22] and EV-miRNA sequencing,[19] ensuring reliable miRNA detection while minimizing technical variability.

To evaluate its accuracy, we analyzed pEV samples from three patients with active disease (both pre- and on-treatment), three samples from patients that responded to their treatment (partial response [PR] or better) according to International Myeloma Working Group (IMWG)-response criteria (see STAR Methods for more details) and three healthy controls. EVs were isolated from 1mL EDTA plasma by standardized SEC using qEV^{tm} columns, and tunable resistive pulse sensing measurements revealed mean particle concentrations ranged from 2.5E10 particles/mL in patients with active disease, 3.6E10 particles/mL in samples of patients with response to treatment, and 5.8E10 particles/mL in healthy donors (Figures 1C and S1A). Adhering to the most recent MISEV (minimal information for studies of extracellular vesicles) guidelines,[23] western blotting showed typical EV-enriched protein markers CD63, CD81, flotillin 1, and syntenin, while the ER-associated protein calnexin could not be detected in EV enriched fractions (Figure S1B). Electron microscopy revealed a mixed population of predominantly small EVs alongside a smaller population of larger EVs (Figure S1C).

To examine the cellular origin of circulating EVs in our patient samples, we isolated and sequenced total RNA from EVs of patients with active disease and from patients with a confirmed response to treatment. We then conducted transcriptome alignment followed by deconvolution using CIBERSORT and the LM22 single-cell sequence reference dataset,[24] which comprises 22 immune cell types. This analysis revealed a diverse array of potentially immune-cell-derived RNAs within the bulk EV population. However, quantitative differences between active and responsive disease states could not be determined with this method (Figure 1D).

Having established that the level of circulating EVs in MM patients itself does not appear to be associated with response status, we next assessed the biomarker potential of pEV-isomiRs. To this end, we used our recently developed IsoSeek small RNA sequencing method, optimized for limited input material.[17,19] To validate IsoSeek's robustness in profiling pEV isomiRs, we compared its performance to the standard NEBNext protocol (see detailed comparison in STAR Methods). IsoSeek demonstrated superior accuracy in pEV isomiR detection, as evidenced by higher proportion of the miRNA class mapped reads relative to other RNA species (Figure S2J). Additionally, the single-nucleotide resolution offered by IsoSeek allowed for isomiR accurate profiling, increasing the number of features from approximately 400 miRNAs (classical miRNA annotation) to 15,000 isomiRs in each plasma EV sample, with around 1,000 of these having more than 10 read counts per million (Figures S2A and S2B). Overall, the results underscore Isoseek's

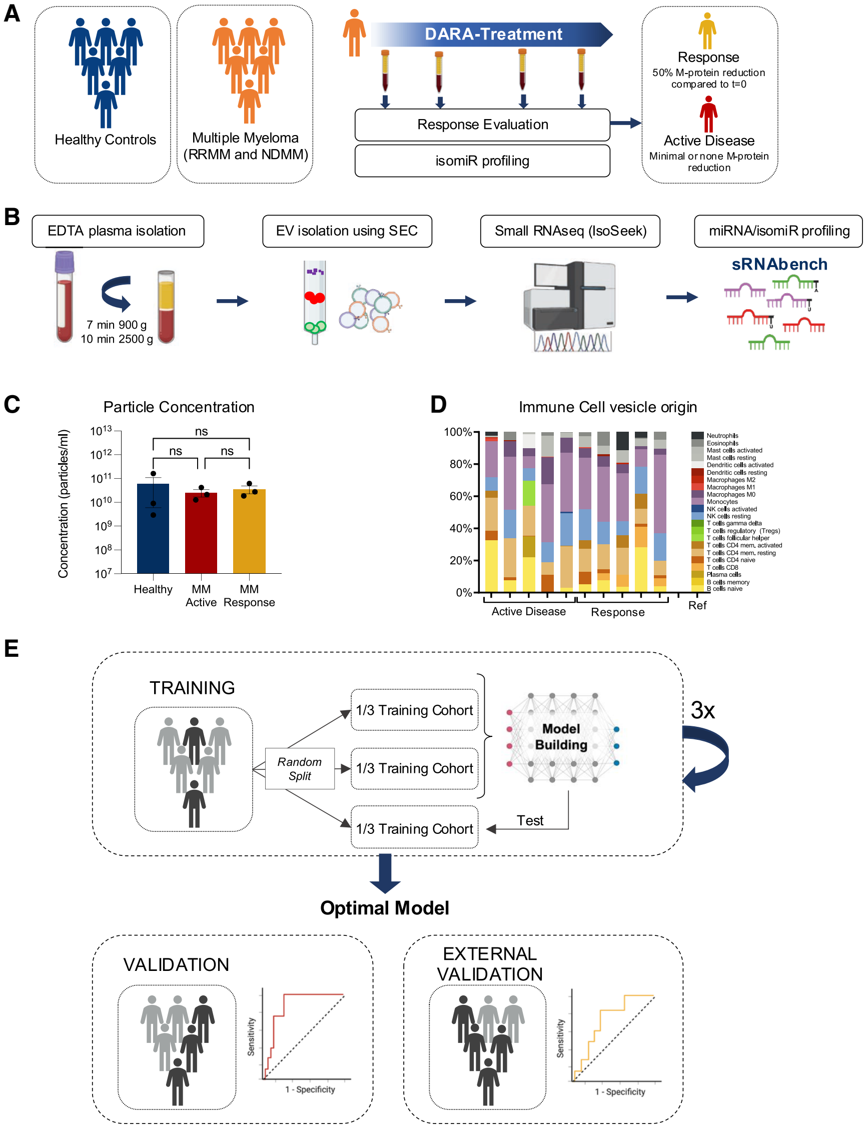

Figure 1. Schematic workflow overview and MM pEV characterization

(A) Simplified overview of the patients included in the different models.

(B) Schematic overview of the workflow for plasma extracellular vesicles (pEVs) isolation, followed by EV characterization and miRNA sequencing and subsequent analyses.

(C) Particle concentration of plasma EVs from healthy donors and MM patients with active disease or clinical response using Exoid. Data are shown as the mean ± standard error of the mean (SEM) (n = 3).

(D) Deconvolution (CIBERSORTx) of mRNA sequencing data from EVs from 5 patients with active MM and 5 patients responding to treatment, using LM22 single-cell sequencing dataset as reference. No clear difference can be observed between the two groups, and almost all immune cell types are represented in both.

(E) Schematic overview of the model building procedure using a cross-validated approach and validation in never-seen datasets. Figure was partially created with BioRender.com.

enhanced accuracy for obtaining plasma EV miRNA/isomiR profiles for biomarker purposes.

A pEV-miRNA classifier signature differentiates MM patients with active disease from age-/gender-matched healthy individuals

To investigate the diagnostic potential of pEV-associated miRNAs and isomiRs in MM, we performed IsoSeek miRNA profiling on a total of 40 samples (see Table S1). The cohort included 34 MM patients with progressive disease (PD) after 1 or more prior treatment lines, alongside 44 age- and gender-matched healthy controls.

Principal-component analysis (PCA) revealed no clear clustering of samples from patients with active disease and samples from healthy donors, suggesting that the relative abundance of a single miRNA does not distinguish the two subgroups (Figure S3A). Similarly, relapsed/refractory MM (RRMM) patients receiving either nivolumab-daratumumab (NIVO-DARA) or daratumumab-all trans retinoic acid (DARA-ATRA) combination treatment could not be distinctly separated (Figure S3B).

To develop a robust miRNA-based classifier that differentiates patients with active MM from healthy controls, we randomly split the 84 samples into a training set (n = 51, 2/3 of total) and a validation set (n = 51, 1/3 of total). We applied cross-validated LASSO regression on the training set to minimize feature selection bias and avoid overfitting (see Figure 1E for schematic overview of the model building process). These steps are critical when using machine learning on data where the number of features i.e., miRNAs and isomiRs, exceed the sample (cohort) size.[14,25]

We evaluated two different miRNA annotations as feature input: (1) a "classic miRNA annotation," where all reads of a given miRNA including its and derivate sequences are aggregated, regardless of any known biological significance of those derivates and (2) a functional "isomiR annotation," where functionally verified canonical miRNAs and non-templated additions (NTA) NTA-A and NTA-U are considered as independent features (see STAR Methods for details).

Using classic miRNA annotation, we identified an optimal 22-miRNA model with the lowest misclassification error in the training set (area under the curve [AUC] = 1.0). This model achieved a highly discriminatory performance with an AUC of 0.98 (95% CI: 0.94–1.00) in the independent validation set (n = 33), with only three misclassifications (Figure 2B, blue line). Using isomiRs as input features, a larger model

Table 1. Characteristics of patients with MM and healthy individuals

MM patients overview	49 patients
Gender	4 male<br>25 female
Age	median: 62 years<br>range: 34–87 years
Treatment line	median: 3<br>range: 0-11
Treatment regimen (treatment at time of sample collection)	
**RRMM daratumumab trial patients**	**30 patients**
NIVO-DARA +/− low-dose cyclophosphamide	15 patients
DARA-ATRA	15 patients
**Biobank (Biolymph VUmc/AMC)**	**19 patients (17/2)**
NDMM	9 patients
VTd followed by HDM/ASCT	3
VCd followed by HDM/ASCT	2
Lenalidomide maintenance after HDM/ASCT	2
KTd followed by HDM/ASCT (CarthaDex trial)	1
KRd followed by HDM/ASCT (Hovon503 trial, arm B)	1
RRMM	10 patients
Daratumumab (+/− lenalidomide/dexamethasone)	4
Standard of care CARTITUDE-4 trial	1
TRIMM-2 trial	1
VCd followed by DLI	1
PCd	1
EPd	1
DVd	1
Monoclonal protein	
IgG kappa	28
IgG lambda	4
IgA kappa	2
IgA lambda, non/oligo-secretor	3
IgM kappa	1
FLC kappa	10
FLC lambda	1
High-risk cytogenetic abnormality	
High risk	19
Standard risk	10
Unknown	20

Healthy donors	*N* = 48
Gender	24 male<br>24 female
Age	median: 64 years<br>range: 45–78 years

HDM, high-dose melphalan; ASCT, autologous stem cell transplantation; NIVO, nivolumab; DARA, daratumumab; ATRA, all trans retinoic acid; VTd, bortezomib-thalidomide-dexamethasone; VCd, bortezomib-cyclophosphamide-dexamethasone; KTd, carfilzomib-thalidomide-dexamethasone; KRd, carfilzomib-lenalidomide-dexamethasone; standard of care CARTITUDE-4, Arm, either bortezomib or daratumumab, with pomalidomide and dexamethasone; TRIMM-2 trial, talquetamab-daratumumab-pomalidomide; DLI, donor lymphocyte infusion; PCd, pomalidomide-cyclophosphamide-dexamethasone, EPd, elotuzumab-pomalidomide-dexamethasone; DVd, daratumumab-bortezomib-dexamethasone. Based on the criteria proposed by Sonneveld et al. Blood 2016, high risk cytogenetics is defined by the presence of t(4;14), t(14;16), t(14;20), del(17/17p), and/or gain(1q).

CellPress
OPEN ACCESS

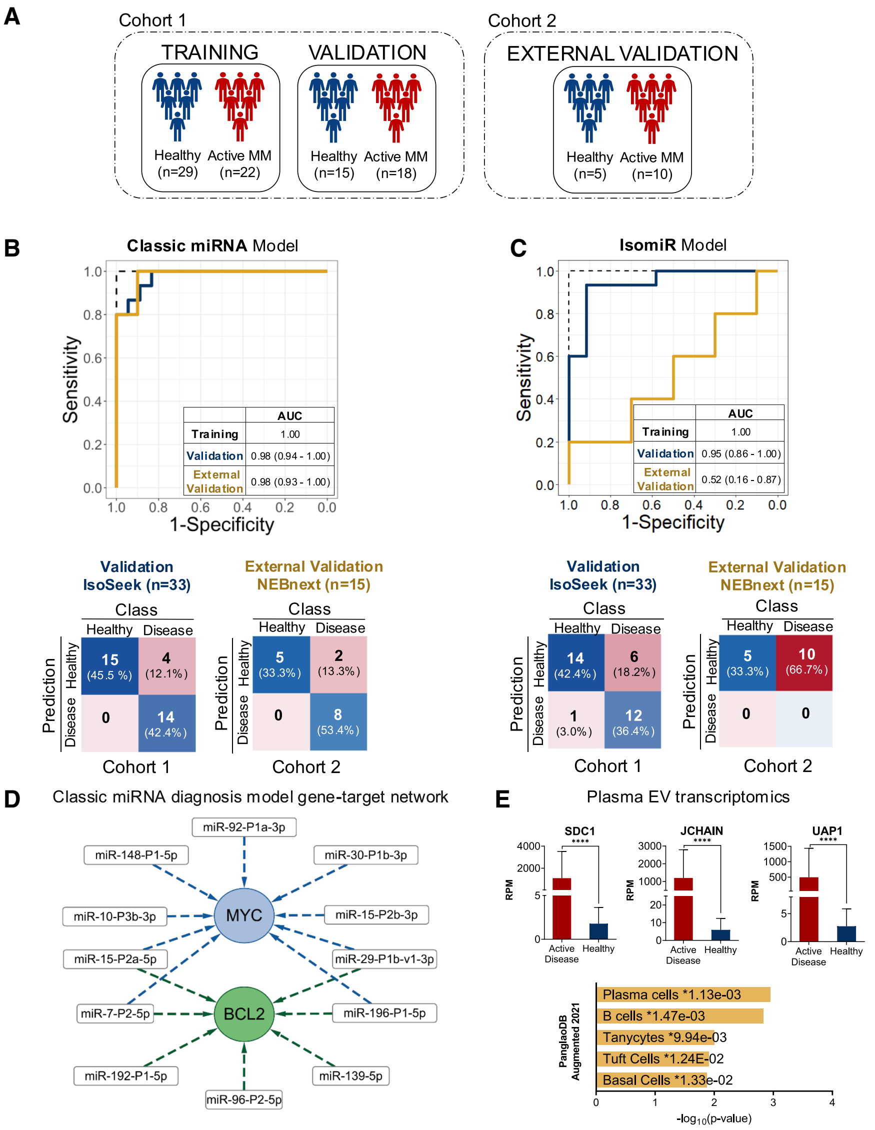

Figure 2. A miRNA network in plasma EVs distinguish patients with active MM from healthy controls

(A) Schematic overview of the cohorts used for model building (cohort 1), model validation (cohort 1), and external validation (cohort 2), including sample sizes.

(B) ROC curves of the classic miRNA disease detection model. In blue, ROC curve of the validation set, and in yellow, ROC curve of the external validation. AUC and confidence intervals are shown in the table. The model validates with a high AUC in both validation sets. Below are the confusion matrix of the two validations sets (internal on the left, external on the right), for the classic miRNA model. Both matrices show a high true positive rate and a low false negative rate.

(C) ROC curves of the isomiR disease detection model. In blue, ROC curve of the validation set, and in yellow, ROC curve of the external validation. AUC and confidence intervals are shown in the table. The model achieves a high AUC in the internal validation set but not in the external set. Below are the confusion matrix of the two validations sets (internal on the left, external on the right), for the isomiR model. Only the internal validation set shows a high true positive rate and a low false negative rate.

(D) Reduced network of the miRNA-gene-targets in the classical miRNA diagnosis model, which includes MYC and BCL2.

(E) Top: visualization of the RPM levels of three of the top differentially expressed mRNAs (SDC1, JCHAIN, and UAP1) in patients with MM AD compared to healthy individuals. Data are shown as the mean ± standard error of the mean (SEM) ($n = 5$ per group). Asterisks (*) denote statistically significant differences: ****$p < 0.0001$. as determined by t test. Bottom: PanglaoDB Augmented 2021 enriched cell types in the AD samples compared to healthy individuals, with plasma cells showing the highest significant enrichment.

with 39 isomiRs was selected based on minimal classification error optimization (AUC = 1.0 in training set), which also achieved a highly discriminatory AUC of 0.95 (95% CI: 0.86–1.00) in the validation set (Figure 2C, blue line) but with two more misclassifications compared to the classic miRNA annotation.

To further evaluate the robustness of the ML-generated classification models, we analyzed external pEV-miRNA sequencing data generated by Manier et al. using the NEBNext protocol on baseline samples from 10 newly diagnosed multiple myeloma (NDMM) patients and 5 healthy controls.[9] Despite considerable pre-analytical (plasma processing and pEV isolation procedure) and analytical differences, our 22 classical miRNA classifier accurately distinguished healthy individuals from patients with active disease (Figure 2B, yellow line) achieving an AUC of 0.98 (95% CI: 0.93–1.00), misclassifying only 2 out of 15 patient samples. In contrast, the isomiR model failed validation in this external patient cohort (AUC: 0.52; 95% CI: 0.16–0.87) (Figure 2C, yellow line). This was not unexpected due to differences in the single-nucleotide resolution and, therefore, relative isomiR quantification when using different protocols for training and validation.[17] Interestingly, when we applied both the miRNA and isomiR models to a disease control cohort of samples from 10 patients with metabolically active (FDG-PET positive) Hodgkin lymphoma (HL) and age-/gender-matched healthy controls ($n = 9$) under identical pre-analytical and analytical conditions, virtually all samples were misclassified (Figures S3C and S3D), indicating disease specificity of the EV-isomiR classifier for MM.

To explore the potential biological relevance of the models trained to detect active MM, we conducted a targetome analysis on the classifier signature. Figure 2D illustrates the most targeted mRNAs by the EV-miRNAs in the best performing diagnostic model (see the complete network in Figure S3E), which included MYC and BCL2, established druggable MM targets.[5] Thus, bulk

pEVs from MM patients with active disease may contain a "tumor-derived signal" that originates, at least partly, from clonally expanding plasma cells. To test this hypothesis, we performed EV-mRNA sequencing on pEVs from 5 patients with active disease and 5 healthy controls. Gene set enrichment analysis (GSEA) revealed a significant enrichment for plasma cells and B cell ontology in active MM samples (Figure 2E, bottom). This unbiased approach was further supported by differential expression (DE) (in this case transcript abundance in pEVs) analysis, which identified several plasma cell specific transcripts as enriched in the total pEV pool isolated from patients with active disease (Figure 2E, top).

Collectively, these findings suggest that pEVs from MM patients with active disease contain miRNAs, isomiRs, and mRNAs originating from malignant plasma cells and tumor-associated stroma in the BM that may be leveraged for liquid biopsy in MM patients.

EV-isomiRs as biomarkers for monitoring disease activity in patients with MM

Currently, MM diagnosis relies on invasive bone-marrow biopsies, while treatment response is monitored through M-protein or free light chain levels in biofluids. Although sensitive, these markers provide limited insight into tumor evolution and lack substantial prognostic value. To investigate whether pEV-miRNAs could be used as a dynamic monitoring tool, we applied IsoSeek to 70 plasma samples from 24 RRMM patients enrolled in two different clinical trials that employed daratumumab-containing combination therapy (see Table S2 for details).

We randomized the 70 samples (35 from patients with active disease and 35 from patients who had achieved a PR or better per IMWG criteria at time of collection) into a training set (n = 42) and a validation set (n = 28). PCA analysis of the total set revealed no clear separation between samples from patients with response or active disease (Figure S4A), and also no separation based on type of daratumumab-based treatment regimen (Figure S4B). Using our cross-validated LASSO regression model strategy (Figure 1E), we developed two models: one using the classic miRNA annotation and another using isomiR classification. The classic miRNA model, consists of only 3 miRNAs and yielded a very promising performance for response assessment, achieving an AUC of 0.92 in the independent validation samples from RRMM patients (95% CI: 0.80–1.00) (Figure 3B, blue line). The isomiR model (Figure 3C, blue line), which included 12 isomiRs (6 canonical miRNAs, 3 NTA-U, and 3 NTA-A isomiRs), achieved a slightly higher AUC of 0.93 (95% CI: 0.81–1.000) in the validation set, misclassifying two samples as "response" while M-protein dynamics indicated active disease.

To investigate the isomiR model's specificity to assess response beyond trials evaluating daratumumab-based regimens, we prospectively collected samples from 13 RRMM patients and 4 NDMM patients treated with standard of care (SOC) regimens or within a different clinical trial (Table S2). Despite differences in treatment, most samples were correctly classified, and the isomiR model outperformed the classic miRNA model (AUC: 0.77; 95% CI: 0.52–1.00 for the classical model) with an AUC of 0.98 (95% CI: 0.94–1.00) in this independent validation set (Figure 3C, yellow line). Although two "response" samples were misclassified, all 5 patients receiving daratumumab-containing regimens outside the clinical trials were correctly classified.

To gain more biological insights into the isomiR classifier, we conducted a targetome analysis of all isomiRs in the model, identifying as targets TLR4 and CDK6 that may be clinically relevant (Figure 3D; Figures S4E and S4F).[26,27] Additionally, we wished to assess whether implementing our sequencing-based approach may be translatable to diagnostic methods more commonly used in clinical practice. We re-sequenced 10 samples (5 from patients with active disease and 5 from patients undergoing response to treatment [PR or better]) using the MiSeq Illumina sequencing platform. Both the classical (Figure S4C) and isomiR models (Figure S4D) correctly classified most samples, with a few misclassifications aligning with those samples near the threshold obtained on the Novaseq platform.

Finally, we sought to validate the robustness of the response model in serial samples from 9 RRMM patients undergoing daratumumab-containing treatment, comparing the response status as predicted by the pEV-isomiR model (from Figure 3C) to the gold standard for response assessment M-protein metric (Figure 4; Figure S5). The pEV-isomiR response model closely mirrored M-protein-based response dynamics during treatment, with only a few misclassifications (Figures 5A and 5D). Interestingly, however, the pEV-isomiR model misclassified two patients as having a "response" (indicated by arrows), whereas M-protein levels indicated "active disease." Yet, longitudinal follow-up revealed that both patients eventually achieved a partial or better response several months later, suggesting that, in some cases, pEV isomiRs may be able to not only monitor but also predict response.

Overall, these findings highlight pEVs as a biologically relevant, minimally invasive source of isomiRs that have biomarker potential as response assessment tool for RRMM patients.

Plasma EV-isomiRs are prognostic biomarkers for survival in multiple myeloma

To explore the prognostic potential of pEV-isomiRs in predicting durable responses for individual patients, we analyzed pre-treatment samples from 34 MM patients with active disease that were to receive a daratumumab-containing regimen. We adhered to a six-months threshold based on prior studies aimed at predicting durable response in MM patients.[4] For the training set, we included samples from 19 RRMM patients who failed at least two prior treatment lines (see Table 2). Among these patients, 8 achieved a durable response (PR or better for more than six months) to daratumumab, while 11 were refractory or experienced an early relapse (Figure 5A). The validation set comprised samples from 10 RRMM patients before receiving daratumumab-containing regimens and 5 NDMM of which 4 received SOC (VRD or VTd induction/Vtd followed by autologous stem cell transplantation and lenalidomide maintenance). Of the 15 patients in the validation set, 7 exhibited poor responses to treatment (i.e., PD within 6 months of treatment, all RRMM patients) and 8 had durable response (3 RRMM and 5 NDMM patients, see Table 2).

An isomiR model (containing 12 isomiRs) achieved a promising AUC of 0.84 in the validation set (95% CI: 0.63–1.00, Figure 5B, left), misclassifying only three patients: two NDMM

CellPress
OPEN ACCESS

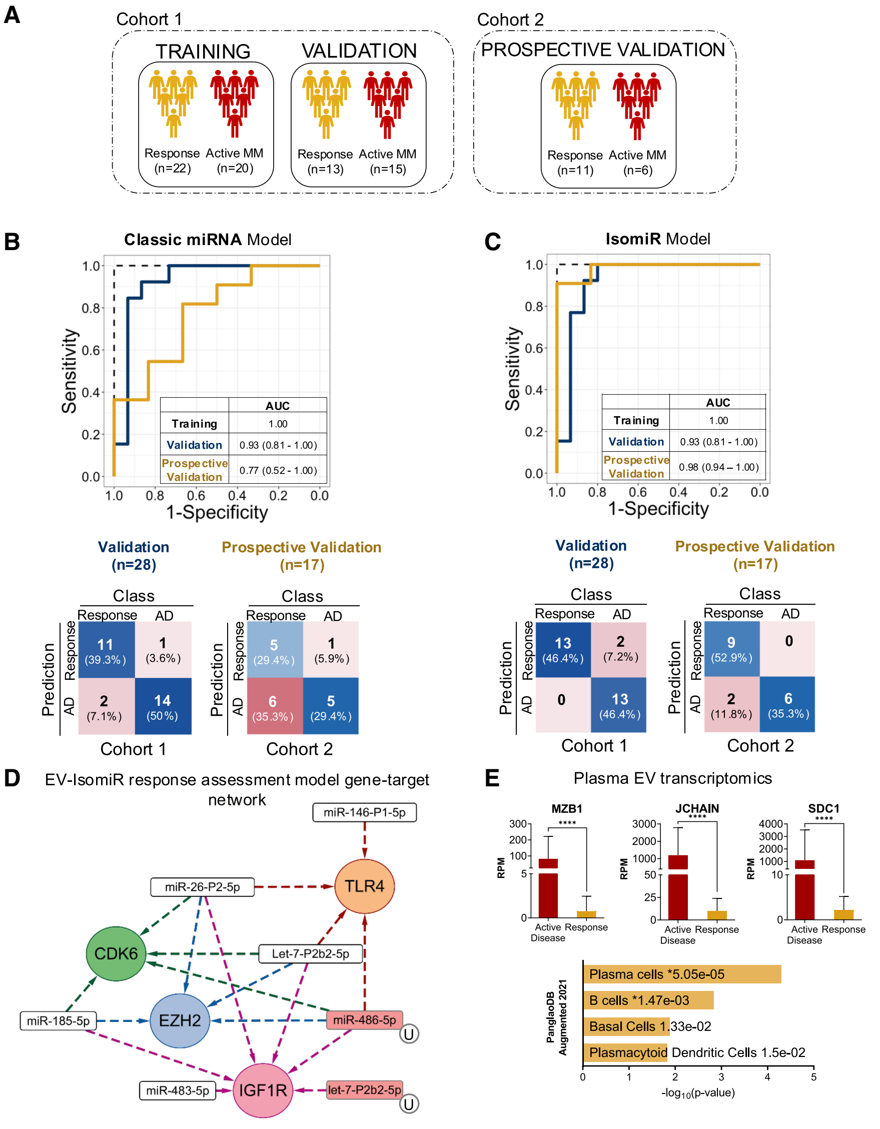

Figure 3. pEV-IsomiRs for on-treatment response assessment in patients with multiple myeloma

(A) Schematic overview of the cohorts used for model building (cohort 1), model validation (cohort 1), and prospective validation (cohort 2), including sample sizes.

(B) ROC curves of the classic miRNA response assessment model. In blue, ROC curve of the validation set, and in yellow, ROC curve of the prospective validation set. AUC and confidence intervals are shown in the table. The model validates with a good AUC in the retrospective data (AUC: 0.89) and worse in the prospective dataset (AUC: 0.77). Below are the confusion matrix of the two validations sets (retrospective on the left, prospective on the right), for the classic miRNA model.

(C) ROC curves of the isomiR response assessment model. In blue, ROC curve of the retrospective validation set, and in yellow, ROC curve of the prospective validation set. AUC and confidence intervals are shown in the table. The model achieves a high AUC in both sets (AUC: 0.90 in the retrospective and AUC: 0.98 in the prospective). Below are the confusion matrix of the two validations sets (retrospective on the left, prospective on the right), for the isomiR model.

(D) Reduced network of the miRNA-gene-targets in the isomiR response assessment model.

(E) Top: visualization of the RPM levels of three of the top differentially expressed mRNAs (MZB1, JCHAIN, and SDC1) in MM patients with active disease, compared to patients with MM responding to treatment (responders). Data are shown as the mean ± standard error of the mean (SEM) (n = 5 per group). Asterisks (*) denote statistically significant differences: ****$p < 0.0001$. as determined by t test. Bottom: PanglaoDB Augmented 2021 enriched cell types in the active disease samples compared to responders, with plasma cells showing the highest significant enrichment.

patients who received SOC and only one RRMM patient who received daratumumab. This yielded a positive predictive value (PPV) of 75% and a negative predictive value (NPV) of 86% (Figure 5B, right). The classic miRNA model did not achieve a good performance (Figure S6D), highlighting the importance of the isomiR profiling.

Interestingly, the isomiR-gene-target network included BCL2 and MYC as targets (Figure 5C, left), in agreement with the active MM model network in Figure 2D. Kaplan-Meier analysis of PFS, including all RRMM patients from the train and validation set (n = 29) achieved a hazard ratio (HR) of 33.1 (95% CI: 4.2–262, $p < 0.005$). Despite the limited number of patients and follow-up period, the durability of response classifier signature was also predictive for OS yielding an HR: 3.8 (95% CI: 1.05–13.99, $p < 0.05$). Kaplan-Meier analysis of PFS of the full cohort including NDMM achieved an HR of 9.04 (Figure S6A) and an HR of 3.61 for OS (Figure S6B).

Among the miRNAs in the model, canonical mir-148-P1-3p exhibited a set of biologically relevant targets, including the already mentioned BCL2 and MYC. To explore its tissue specificity, we assessed its abundance across all annotated tissue and cell sorted samples in IsomiRDB (n = 1692)[28] as well as all “plasma cell” samples from miSRA profiler.[29] Results indicated that miR-148-P1-3p expression is highly specific to plasma cells compared to other cell types and tissues.

Comparison of baseline M-protein levels with the isomiR-based prediction model (Figure S6C) highlights the superior predictive value of the model, as further supported by Kaplan-Meier analysis of progression-free and OS (Figures S6E and S6F).

These findings suggest that, if verified in large(r) cohorts with longer follow-up, an EV-isomiR test could support clinicians in selecting RRMM patients most likely to benefit from specific (daratumumab-containing) treatments, while potentially sparing others from ineffective interventions.

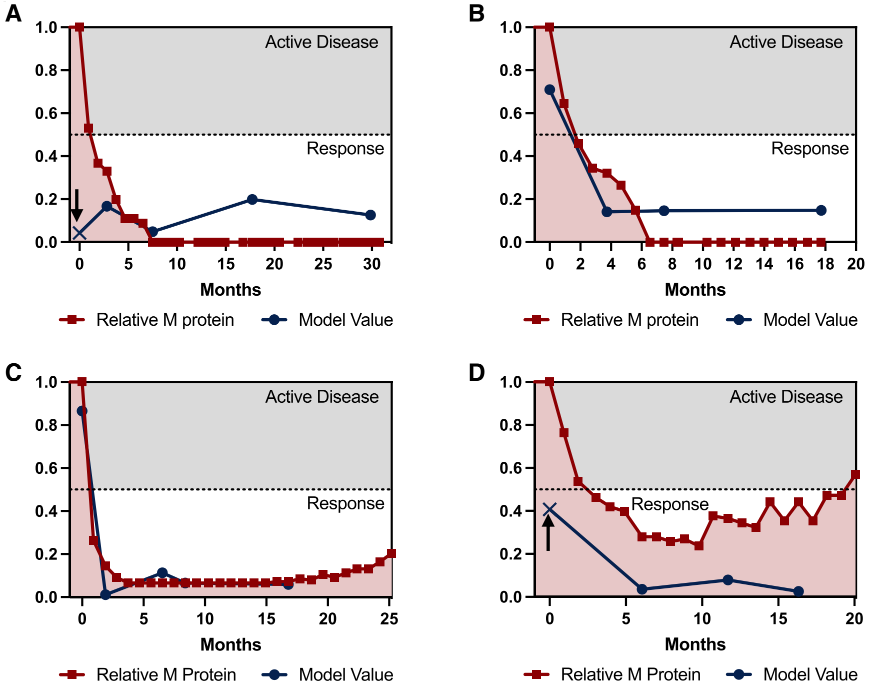

Figure 4. pEV-IsomiRs allow response monitoring in individual MM patients over time

(A–D) pEV-isomiR model predictions over time (blue lines, each blue dot represents a measurement) compared to M-protein levels (red lines, each red dot represents a measurement) for four individual MM patients, showing that the model closely tracks the M-protein metric, demonstrating its robustness. Although some misclassifications are present (indicated by the arrows), the model levels in those samples were consistent with the status of the patients in the future months. M-protein relative level (right axis) at each time point was calculated as the ratio at that time point as compared to M protein level at the beginning of treatment.

DISCUSSION

Despite an advancing treatment landscape, MM remains an incurable disease, with initial remissions often followed by multiple relapses.[1] Resistance is driven by a significant degree of genomic heterogeneity and the protective BM microenvironment. To minimize toxicity and improve outcomes in patients with MM, there is a critical need for easily accessible biomarkers that can not only report, but ideally predict, which patients are likely to benefit from treatment.[5,6,30]

In this study, we applied a scalable protocol for EVs isolation from 1 mL of plasma samples collected from RRMM patients receiving immunotherapy. Using IsoSeek, a high-resolution small RNA sequencing method, we generated machine learning-based response and outcome prediction models based on biologically motivated isomiR signatures.

Multiple immune defects contribute to disease progression and immunotherapy failure.[1] EVs released by the bone-marrow tumor microenvironment (TME) and malignant plasma cells may capture key unique features of MM pathology.[31–34] The MM TME is highly relevant for predicting outcomes to immunomodulatory drugs.[5] Upon secretion by living cells, EVs are short-lived while they protect their RNA content from degradation, thus representing a dynamic source of minimally invasive biomarkers.[13,35] Moreover, EV-miRNA biomarkers are more stable than free circulating miRNAs or ctDNA due to the protective vesicular structure of EVs, and they carry a rich array of molecules, enabling a more comprehensive analysis as compared to targeted methods such as blood-based mass spectrometry.

We found that a significant proportion of EVs in the circulation of MM patients with active disease are of immune cell origin (Figure 2D), and although their exact origin cannot be determined, DE analysis suggested that patients with active MM have increased levels of plasma cell related transcripts in circulation, further validated by unbiased, gene set enrichment analysis (GSEA) (Figures 3F and 4F). These finding suggests that a proportion of circulating EVs in MM patients likely originates from malignant plasma cells in the BM, which is in agreement with recent EV transcriptomics observations in prostate cancer patients with bone metastasis.[36]

Previous studies have shown that individual EV-bound miRNAs, measured by RT-qPCR, have prognostic value for NDMM patients treated with bortezomib/dexamethasone, high-dose melphalan and autologous stem-cell transplantation.[9] In this study, we focused on the diagnostic potential of EV-bound isomiRs in RRMM patients receiving daratumumab-containing regimens. Using cross-validated, logistic regression and penalization/shrinkage of the parameters (LASSO), we built robust classifiers, a method that has shown strength in validation of miRNA signatures from "noisy" data and smaller sized patient cohorts,[14] thus enhancing prediction accuracy and interpretability of the statistical models. IsomiR signatures showed high accuracy in "never seen" data (i.e., samples not part of the training set), as compared to models with classic miRNA annotation. Notably, classic miRNA annotation correctly classified the patient samples used in the Manier study.[9] It was reassuring to observe that let-7b-5p, which was prognostic for NDMM patients in the Manier study, was also part of our LASSO-based signature. The combination of functional isomiRs in our models achieved high accuracy for response prediction, PFS and to a lesser extent OS, which may be related to the limited follow up. Interestingly, serial analysis suggested that EV-isomiR models may predict future response status better than M-protein levels, possibly because of the extended half-life of M-proteins of the IgG subtype compared to circulating EVs.[37]

Although establishing a direct link between EV-miRNAs and MM tumor niches in the BM is not possible, isomiRs in our classification signatures (Figure 3E) target MYC or BCL2 oncogenes driving myelomagenesis.[5] While not established, a recent

CellPress
OPEN ACCESS

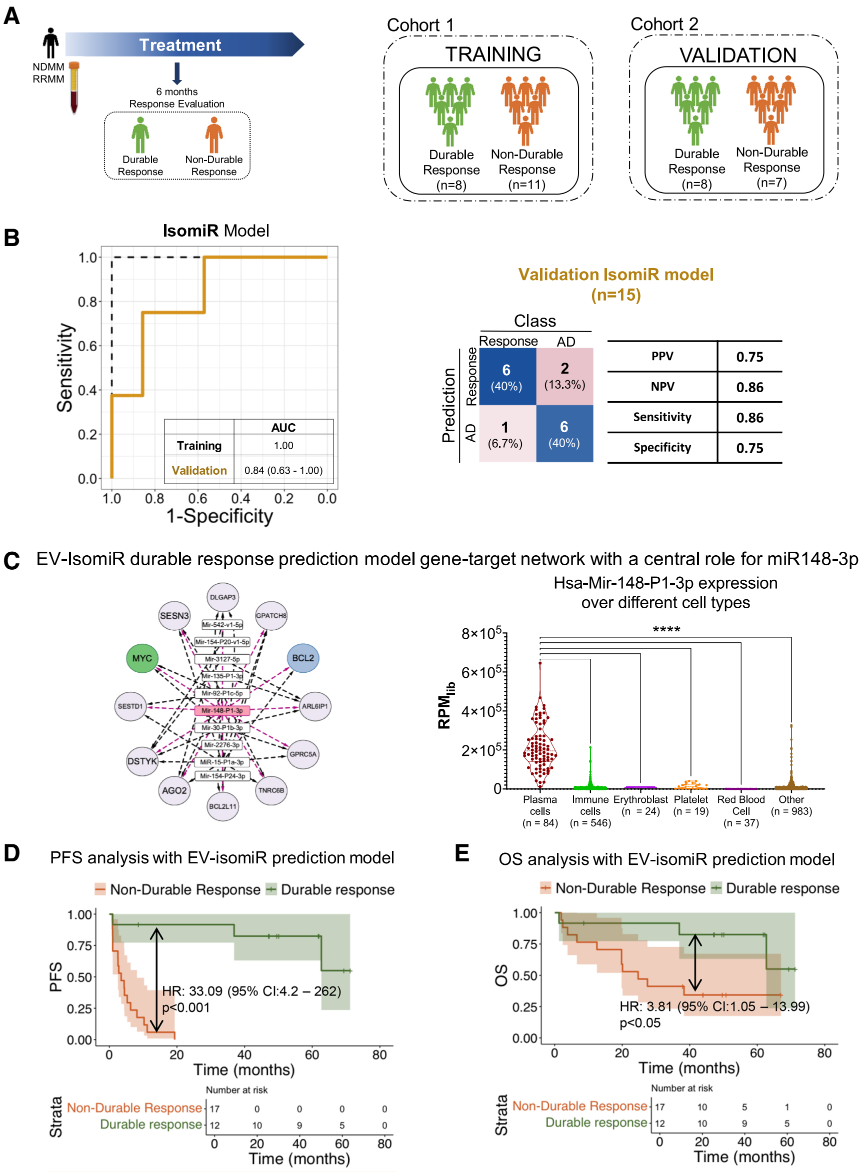

Figure 5. Pre-treatment pEV-isomiR model forecast durable response, PFS and OS

(A) Schematic overview of the patient group generation (left) and the cohorts used for model building (cohort 1) and model validation (cohort 2), including sample sizes.

(B) (Left) ROC curve of the isomiR durable response prediction model. The model achieved a high AUC of 0.84 in the validation set. (Right) Confusion matrix of the validation set for the isomiR model and sensitivity, specificity, PPV, and NPV of the model.

(C) (Left) Reduced network of the miRNA-gene-targets in the isomiR durable response model. (Right) Expression of Has-Mir-148-P1-3p over different cell types, including plasma cells. All samples with a tissue/cell type annotation in IsomiRDB were included, and number of samples in each category is summarized below each violin plot (n). Asterisks (*) denote statistically significant differences: **** $p < 0.0001$. as determined by t test.

(D) Progression-free survival (PFS) of the RRMM patients ($n = 29$). The survival curve was computed using the Kaplan-Meier method. The hazard ratio (HR) calculated by Cox-regression is 33.09 (CI: 4.2–262, $p < 0.0001$).

(E) Overall survival (OS) analysis of the RRMM patients ($n = 29$). The survival curve was computed using the Kaplan-Meier method. The HR calculated by Cox-regression is 3.81 (CI: 1.05–13.99, $p < 0.05$).

CRISPR screen suggests that indirect targeting of MYC in MM has promise that may overcome drug resistance.[5,38,39] In addition, plasma cell mRNA enrichment (Figures 3F and 4F) was determined in MM patients with active disease. Several miRNAs in our signatures are of particular biological interest, such as miR-221, an oncomiR implicated in *in vitro* drug resistance to dexamethasone and melphalan and miR-324-5p, located on chromosome 17p, which is often deleted in MM and suppresses proliferation and enhances bortezomib sensitivity in MM cells.[40,41] The “durable response” classification signature is centered around miR-148-3p (Figure 5D), which has an established role in plasma cell biology[42,43] (Figure 5D) and appears linked with an active disease status (Figure 3E). While some miRNAs cannot be directly linked to malignant plasma cells, this is consistent with the observation that multiple immunological traits during immunotherapy determine outcome.[5]

We hypothesize that in MM patients with active disease, miRNA-loaded EVs are also derived from the MM bone-marrow TME and possibly other tissue resident and circulating immune cells. Despite uncertainty on their origin, the miRNA model that distinguishes active MM patients (based on M-protein) from healthy controls appears to have some degree of disease specificity as this model does not classify samples from HL patients. One explanation is that the TME surrounding the proliferating malignant B cells (i.e., BM stroma in MM versus lymph nodes in HL) is different and influences the EV-miRNA signature.

Our study also assessed the prognostic value of EV-miRNAs for RRMM patients. The results showed that modeling functional isomiRs yielded a classifier signature with a high negative predictive value (NPV = 86%; Figure 5B, right). A validated pEV-isomiR blood test could thus help identify RRMM patients that are unlikely to respond to daratumumab therapy, directing them toward novel T cell immunotherapy solutions, such as bispecific antibodies or CAR T cell therapy (chimeric antigen receptor T cell therapy).

Limitations of the study

This translational study was performed with relatively small cohorts from trial- and real-world patients. We aimed to overcome

Table 2. Response status of the training and validation set for durable response, >6 months, prediction model in RRMM and NDMM patients from Figure 5

	Training cohort		Validation cohort	
	Non-durable response	Durable response	Non-durable response	Durable response
Patients with MM included in durable response prediction model (Figure 5)	11 patients<br>11 samples	8 patients<br>8 samples	7 patients<br>7 samples	8 patients<br>8 samples
Gender	6 male<br>5 female	3 male<br>5 female	4 male<br>3 female	3 male<br>5 female
Age	median: 66 years<br>range: 47–80 years	median: 62 years<br>range: 54–87 years	median: 62 years<br>range: 54–71 years	median: 58 years<br>range: 45–77 years
Treatment line	median: 5<br>range: 2-11	median: 4<br>range: 3-8	median: 3<br>range: 2-5	median: 0<br>range: 0-5
Progression free survival (PFS) (in months)	median: 4.3<br>range: 0.9–19.3	median: 24.2<br>range: 7.9–78.5	median: 2.6<br>range: 0.9–10.3	median: 32.2<br>range: 1.4–70.5
Overall survival (OS) (in months)	median: 27.5<br>range: 2.4–67	median: 62<br>range: 8.6–71.2	median: 12.5<br>range: 1.3–50.8	median: 59.4<br>range: 1.4–112
Treatment regimen at time of sample collection				
**RRMM daratumumab trial patients**				
NIVO-DARA +/− low-dose cyclophosphamide	3	5	5	1
DARA-ATRA	8	2	2	2
**Biobank (Biolymph VUmc/AMC)**				
NDMM				
VTd followed by HDM/ASCT	0	0	0	3
VCd followed by HDM/ASCT	0	0	0	1
KTd followed by HDM/ASCT (CarthaDex trial)	0	0	0	1
RRMM				
Daratumumab (+/− lenalidomide/ dexamethasone)	0	1	0	0
Monoclonal protein				
IgG kappa	7	4	5	6
IgG lambda	2	1	1	0
IgA kappa	1	0	0	0
IgA lamda, non/oligo-secretor	0	0	1	0
FLC kappa	0	0	0	1
FLC lambda	1	3	0	1
High-risk cytogenetic abnormality				
High risk	6	4	4	2
Standard risk	2	2	0	5
Unknown	3	2	3	1
Distribution of the samples per analysis group				
**Non-durable response group**	**11**	–	**7**	–
NIVO-DARA (ND) baseline	3	–	5	–
DARA-ATRA (DA) baseline	8	–	2	–
**Durable response group**	–	**8**	–	**8**
NIVO-DARA (ND) baseline	–	5	–	1
DARA-ATRA (DA) baseline	–	2	–	2
Newly diagnosed	–	0	–	5
Daratumumab monotherapy as third line	–	1	–	0

HDM, high-dose melphalan; ASCT, autologous stem cell transplantation; NIVO, nivolumab; DARA, daratumumab; ATRA, all trans retinoic acid; VTd, bortezomib-thalidomide-dexamethasone; VCd, bortezomib-cyclophosphamide-dexamethasone; KTd, carfilzomib-thalidomide-dexamethasone. Based on the criteria proposed by Sonneveld et al. Blood 2016, high risk cytogenetics is defined by the presence of t(4;14), t(14;16), t(14;20), del (17/17p), and/or gain(1q). Second part of the table depicts the sample distribution between the different cohorts.

the limitation in patient numbers, and we designed a machine learning/validation approach that reduces the risk of overfitting. Despite positive results in multiple cohorts, suggesting generalizability of the assay, all patients were treated in one academic center. Further external validation in samples from universally treated patients at different centers will increase the clinical utility. In addition, it has yet to be tested whether our method will yield similar results using different blood tubes than EDTA. If validated, a single EV-isomiR prognostic test may offer an attractive alternative for bone-marrow aspirations or biopsies in addition to (sustained) MRD tests.[3] Nevertheless, MRD negativity is likely to remain the gold standard for predicting long-term outcome. Apart, from the prognostic value for both PFS and OS, EV-isomiR signatures could be trained selectively to help clinicians decide whether a given therapy has a high likelihood of success for individual patients. A potential future "predictive efficacy score" based on the isomiR model could inform clinicians how (un)likely it is that a patient will develop a durable response.

In conclusion, our results suggest that machine learning-derived pEV-isomiR signatures could enhance therapy selection and outcome prediction for RRMM patients. Network analysis on the circulating plasma EV transcriptome may help identify novel drug targets.

RESOURCE AVAILABILITY

Lead contact

Further information and requests for resources should be directed to and will be fulfilled by the lead contact, D. Michiel Pegtel (d.pegtel@amsterdamumc.nl).

Materials availability

This study did not generate new unique reagents.

Data and code availability

- RNA-seq data have been deposited at SRA and are publicly available as of the date of publication. Accession numbers are listed in the key resources table.
- The code generated for this article is available at https://doi.org/10.5281/zenodo.15316217.
- Any additional information required to reanalyze the data reported in this paper is available from the lead contact upon request.

ACKNOWLEDGMENTS

The authors would like to thank Inger Nijhof, Yvonne Jauw, and Patricia W. C. Maas-Bosman for collecting patient samples and Andre Wijfjes and A. Schmitz from GenomeScan BV for technical input and sequencing. We would also like to thank the usage of the computational infrastructure of the Computational Epigenomics Lab of the University of Granada. This work was supported by Stichting Cancer Center Amsterdam (CCA2021-9-77, CCA2023-9-93) to C. G.-M., Spanish Government (AGL2017-88702-C2-2-R) to M.H., multiple grants awarded to D.M.P., including NWO Perspectief Cancer-ID, TKI-health Holland AQrate, and Stichting NEXTGEN HIGHTECH Program (Biomed02).

AUTHOR CONTRIBUTIONS

Conceptualization, C.G.-M., E.E.E.D., M.A.J.v.E., J.R.d.R., and D.M.P.; methodology, C.G.-M., M.A.J.v.E., N.J.G., and D.M.P.; investigation, C.G.-M., E.E.E.D., M.A.J.v.E., N.J.G., S.W., S.A.W.M.V., J.R.T.v.W., E.A.-P., L.B., C.G.M.G.-O., M.H., J.R.d.R., and D.M.P.; writing – original draft, C.G.-M., E.E.E.D., M.A.J.v.E., and D.M.P.; writing – review & editing, all authors; funding acquisition, C.G.-M., J.R.d.R., and D.M.P.; resources, N.W.C.J.v.d.D., M.J.K., K.A.F., C.P.M.V., E.E.E.D., J.M.Z., D.d.J., and D.M.P.

DECLARATION OF INTERESTS

D.M.P. and M.H. were co-founders of Exbiome BV. D.M.P. was CSO of Exbiome BV and served as an advisor for Takeda for which he received travel compensation. D.M.P. received research funding from Gilead, AbbVie (not related to this project), and Amgen (related to this project). C.G.-M. and M.A.J.v.E. received travel compensation from QIAGEN. ExBiome received funding from Amgen for sequencing the samples. Amgen had no role in design of the study and was not involved in the writing of this manuscript. NWCJvdD has received research support from Janssen Pharmaceuticals, Amgen, Celgene, Novartis, Cellectis, and BMS and serves in advisory boards for Janssen Pharmaceuticals, Amgen, Celgene, BMS, Sanofi, Takeda, Roche, Novartis, Bayer, Adaptive, Merck, Kite Pharma, Pfizer, AbbVie, and Servier, all paid to institution.

STAR★METHODS

Detailed methods are provided in the online version of this paper and include the following:

- KEY RESOURCES TABLE
- EXPERIMENTAL MODEL AND STUDY PARTICIPANT DETAILS
  - Clinical plasma samples processing and legislation
- METHOD DETAILS
  - Definitions of the clinical sample groups
  - Plasma extracellular vesicle isolation
  - Western blot
  - Transmission Electron microscopy
  - Particle measurement
  - RNA isolation and quality control
  - Small RNA library preparation and sequencing
  - Total RNA library preparation and sequencing
- QUANTIFICATION AND STATISTICAL ANALYSIS
  - Processing of sequencing data and microRNA profiling
  - Processing and analysis of total RNA sequencing data
  - IsoSeek performance evaluation on plasma extracellular vesicle samples
  - Statistical modeling
  - MicroRNA target network analysis
  - Survival analysis
  - Deconvolution analysis
- ADDITIONAL RESOURCES

SUPPLEMENTAL INFORMATION

Supplemental information can be found online at https://doi.org/10.1016/j.xcrm.2025.102358.

Received: December 3, 2024
Revised: April 3, 2025
Accepted: August 19, 2025
Published: September 16, 2025

REFERENCES

1. van de Donk, N.W.C.J., Pawlyn, C., and Yong, K.L. (2021). Multiple myeloma. Lancet (London, England) *397*, 410–427. https://doi.org/10.1016/S0140-6736(21)00135-5.
2. Burgos, L., Puig, N., Cedena, M.-T., Mateos, M.-V., Lahuerta, J.J., Paiva, B., and San-Miguel, J.F. (2020). Measurable residual disease in multiple myeloma: ready for clinical practice? J. Hematol. Oncol. *13*, 82. https://doi.org/10.1186/s13045-020-00911-4.
3. Bustoros, M., Mouhieddine, T.H., Detappe, A., and Ghobrial, I.M. (2017). Established and Novel Prognostic Biomarkers in Multiple Myeloma. Am. Soc. Clin. Oncol. Educ. Book *37*, 548–560. https://doi.org/10.1200/EDBK_175175.

4. San-Miguel, J., Avet-Loiseau, H., Paiva, B., Kumar, S., Dimopoulos, M. A., Facon, T., Mateos, M.-V., Touzeau, C., Jakubowiak, A., Usmani, S. Z., et al. (2022). Sustained minimal residual disease negativity in newly diagnosed multiple myeloma and the impact of daratumumab in MAIA and ALCYONE. Blood *139*, 492–501. https://doi.org/10.1182/blood.2020010439.
5. Larrayoz, M., Garcia-Barchino, M.J., Celay, J., Etxebeste, A., Jimenez, M., Perez, C., Ordoñez, R., Cobaleda, C., Botta, C., Fresquet, V., et al. (2023). Preclinical models for prediction of immunotherapy outcomes and immune evasion mechanisms in genetically heterogeneous multiple myeloma. Nat. Med. *29*, 632–645. https://doi.org/10.1038/s41591-022-02178-3.
6. Anderson, K.C., Auclair, D., Adam, S.J., Agarwal, A., Anderson, M., Avet-Loiseau, H., Bustoros, M., Chapman, J., Connors, D.E., Dash, A., et al. (2021). Minimal Residual Disease in Myeloma: Application for Clinical Care and New Drug Registration. Clin. Cancer Res. *27*, 5195–5212. https://doi.org/10.1158/1078-0432.CCR-21-1059.
7. Mithraprabhu, S., Reynolds, J., Turner, R., Quach, H., Horvath, N., Kerridge, I., Kalff, A., Bergin, K., Hocking, J., Yuen, F., et al. (2023). Circulating tumour DNA analysis predicts relapse and improves risk stratification in primary refractory multiple myeloma. Blood Cancer J. *13*, 25. https://doi.org/10.1038/s41408-023-00796-9.
8. Dutta, A.K., Alberge, J.-B., Lightbody, E.D., Boehner, C.J., Dunford, A., Sklavenitis-Pistofidis, R., Mouhieddine, T.H., Cowan, A.N., Su, N.K., Horowitz, E.M., et al. (2023). MinimuMM-seq: Genome Sequencing of Circulating Tumor Cells for Minimally Invasive Molecular Characterization of Multiple Myeloma Pathology. Cancer Discov. *13*, 348–363. https://doi.org/10.1158/2159-8290.CD-22-0482.
9. Manier, S., Liu, C.J., Avet-Loiseau, H., Park, J., Shi, J., Campigotto, F., Salem, K.Z., Huynh, D., Glavey, S.V., Rivotto, B., et al. (2017). Prognostic role of circulating exosomal miRNAs in multiple myeloma. Blood *129*, 2429–2436. https://doi.org/10.1182/blood-2016-09-742296.
10. Gullà, A., Di Martino, M.T., Gallo Cantafio, M.E., Morelli, E., Amodio, N., Botta, C., Pitari, M.R., Lio, S.G., Britti, D., Stamato, M.A., et al. (2016). A 13 mer LNA-i-miR-221 Inhibitor Restores Drug Sensitivity in Melphalan-Refractory Multiple Myeloma Cells. Clin. Cancer Res. *22*, 1222–1233. https://doi.org/10.1158/1078-0432.CCR-15-0489.
11. Drees, E.E.E., Roemer, M.G.M., Groenewegen, N.J., Perez-Boza, J., van Eijndhoven, M.A.J., Prins, L.I., Verkuijlen, S.A.W.M., Tran, X.-M., Driessen, J., Zwezerijnen, G.J.C., et al. (2021). Extracellular vesicle miRNA predict FDG-PET status in patients with classical Hodgkin Lymphoma. J. Extracell. Vesicles *10*, e12121. https://doi.org/10.1002/jev2.12121.
12. Arroyo, J.D., Chevillet, J.R., Kroh, E.M., Ruf, I.K., Pritchard, C.C., Gibson, D.F., Mitchell, P.S., Bennett, C.F., Pogosova-Agadjanyan, E.L., Stirewalt, D.L., et al. (2011). Argonaute2 complexes carry a population of circulating microRNAs independent of vesicles in human plasma. Proc. Natl. Acad. Sci. USA *108*, 5003–5008. https://doi.org/10.1073/pnas.1019055108.
13. Drees, E.E.E., Groenewegen, N.J., Verkuijlen, S.A.W.M., van Eijndhoven, M.A.J., Ramaker, J., Veenstra, P., Hussain, M., Groothuis-Oudshoorn, C.G.M., de Jong, D., Zijlstra, J.M., et al. (2024). Towards IVDR-compliance by implementing quality control steps in a quantitative extracellular vesicle-miRNA liquid biopsy assay for response monitoring in patients with classic Hodgkin lymphoma. J. Extracell. Biol. *3*, e164. https://doi.org/10.1002/jex2.164.
14. Lindahl, L.M., Besenbacher, S., Rittig, A.H., Celis, P., Willerslev-Olsen, A., Gjerdrum, L.M.R., Krejsgaard, T., Johansen, C., Litman, T., Woetmann, A., et al. (2018). Prognostic miRNA classifier in early-stage mycosis fungoides: development and validation in a Danish nationwide study. Blood *131*, 759–770. https://doi.org/10.1182/blood-2017-06-788950.
15. Rajakumar, T., Horos, R., Jehn, J., Schenz, J., Muley, T., Pelea, O., Hofmann, S., Kittner, P., Kahraman, M., Heuvelman, M., et al. (2022). A blood-based miRNA signature with prognostic value for overall survival in advanced stage non-small cell lung cancer treated with immunotherapy. npj Precis. Oncol. *6*, 19. https://doi.org/10.1038/s41698-022-00262-y.
16. Nishiwada, S., Cui, Y., Sho, M., Jun, E., Akahori, T., Nakamura, K., Sonohara, F., Yamada, S., Fujii, T., Han, I.W., et al. (2022). Transcriptomic Profiling Identifies an Exosomal microRNA Signature for Predicting Recurrence Following Surgery in Patients With Pancreatic Ductal Adenocarcinoma. Ann. Surg. *276*, e876–e885.
17. Gómez-Martín, C., Aparicio-Puerta, E., van Eijndhoven, M.A.J., Medina, J. M., Hackenberg, M., and Pegtel, D.M. (2023). Reassessment of miRNA variant (isomiRs) composition by small RNA sequencing. Cell Rep. Methods *3*, 100480. https://doi.org/10.1016/j.crmeth.2023.100480.
18. Hu, J.F., Yim, D., Ma, D., Huber, S.M., Davis, N., Bacusmo, J.M., Vermeulen, S., Zhou, J., Begley, T.J., DeMott, M.S., et al. (2021). Quantitative mapping of the cellular small RNA landscape with AQRNA-seq. Nat. Biotechnol. *39*, 978–988. https://doi.org/10.1038/s41587-021-00874-y.
19. van Eijndhoven, M.A.J., Scheepbouwer, C., Aparicio-Puerta, E., Hackenberg, M., and Pegtel, D.M. (2023). IsoSeek for unbiased and UMI-informed sequencing of miRNAs from low input samples at single-nucleotide resolution. STAR Protoc. *4*, 102645. https://doi.org/10.1016/j.xpro.2023.102645.
20. Koppers-Lalic, D., Hackenberg, M., Menezes, R.D., Misovic, B., Wachalska, M., Geldof, A., Zini, N., Reijke, T.D., Wurdinger, T., Vis, A., et al. (2016). Noninvasive prostate cancer detection by measuring miRNA variants (isomiRs) in urine extracellular vesicles. Oncotarget *7*, 22566–22578. https://doi.org/10.18632/oncotarget.8124.
21. Scheepbouwer, C., Hackenberg, M., van Eijndhoven, M.A.J., Gerber, A., Pegtel, M., and Gómez-Martín, C. (2023). NORMSEQ: a tool for evaluation, selection and visualization of RNA-Seq normalization methods. Nucleic Acids Res. *51*, W372–W378. https://doi.org/10.1093/nar/gkad429.
22. Bracht, J.W.P., Los, M., van Eijndhoven, M.A.J., Bettin, B., van der Pol, E., Pegtel, D.M., and Nieuwland, R. (2023). Platelet removal from human blood plasma improves detection of extracellular vesicle-associated miRNA. J. Extracell. Vesicles *12*, e12302. https://doi.org/10.1002/jev2.12302.
23. Welsh, J.A., Goberdhan, D.C.I., O'Driscoll, L., Buzas, E.I., Blenkiron, C., Bussolati, B., Cai, H., Di Vizio, D., Driedonks, T.A.P., Erdbrügger, U., et al. (2024). Minimal information for studies of extracellular vesicles (MISEV2023): From basic to advanced approaches. J. Extracell. Vesicles *13*, e12404. https://doi.org/10.1002/jev2.12404.
24. Newman, A.M., Liu, C.L., Green, M.R., Gentles, A.J., Feng, W., Xu, Y., Hoang, C.D., Diehn, M., and Alizadeh, A.A. (2015). Robust enumeration of cell subsets from tissue expression profiles. Nat. Methods *12*, 453–457. https://doi.org/10.1038/nmeth.3337.
25. Lever, J., Krzywinski, M., and Altman, N. (2016). Points of Significance: Model selection and overfitting. Nat. Methods *13*, 703–704. https://doi.org/10.1038/nmeth.3968.
26. Ng, Y.L.D., Ramberger, E., Bohl, S.R., Dolnik, A., Steinebach, C., Conrad, T., Müller, S., Popp, O., Kull, M., Haji, M., et al. (2022). Proteomic profiling reveals CDK6 upregulation as a targetable resistance mechanism for lenalidomide in multiple myeloma. Nat. Commun. *13*, 1009. https://doi.org/10.1038/s41467-022-28515-1.
27. Giallongo, C., Tibullo, D., Camiolo, G., Parrinello, N.L., Romano, A., Puglisi, F., Barbato, A., Conticello, C., Lupo, G., Anfuso, C.D., et al. (2019). TLR4 signaling drives mesenchymal stromal cells commitment to promote tumor microenvironment transformation in multiple myeloma. Cell Death Dis. *10*, 704. https://doi.org/10.1038/s41419-019-1959-5.
28. Aparicio-Puerta, E., Hirsch, P., Schmartz, G.P., Fehlmann, T., Keller, V., Engel, A., Kern, F., Hackenberg, M., and Keller, A. (2023). isomiRdb: microRNA expression at isoform resolution. Nucleic Acids Res. *51*, D179–D185. https://doi.org/10.1093/nar/gkac884.
29. Bioinformatics, C.E., and Hackenberg, M. (2024). bioinfoUGR/miSRA: miSRA Profiler (Zenodo). https://doi.org/10.5281/zenodo.13925083 Version v.1.0.0. .
30. Ubels, J., Sonneveld, P., van Beers, E.H., Broijl, A., van Vliet, M.H., and de Ridder, J. (2018). Predicting treatment benefit in multiple myeloma through

CellPress
OPEN ACCESS

simulation of alternative treatment effects. Nat. Commun. *9*, 2943. https://doi.org/10.1038/s41467-018-05348-5.

31. Laurenzana, I., Trino, S., Lamorte, D., De Stradis, A., Santodirocco, M., Sgambato, A., De Luca, L., and Caivano, A. (2021). Multiple Myeloma-Derived Extracellular Vesicles Impair Normal Hematopoiesis by Acting on Hematopoietic Stem and Progenitor Cells. Front. Med. *8*, 793040. https://doi.org/10.3389/fmed.2021.793040.
32. Yamamoto, T., Nakayama, J., Yamamoto, Y., Kuroda, M., Hattori, Y., and Ochiya, T. (2022). SORT1/LAMP2-mediated extracellular vesicle secretion and cell adhesion are linked to lenalidomide resistance in multiple myeloma. Blood Adv. *6*, 2480–2495. https://doi.org/10.1182/bloodadvances.2021005772.
33. Papanota, A.-M., Karousi, P., Kontos, C.K., Artemaki, P.I., Liacos, C.-I., Papadimitriou, M.-A., Bagratuni, T., Eleutherakis-Papaiakovou, E., Malandrakis, P., Ntanasis-Stathopoulos, I., et al. (2021). A Cancer-Related microRNA Signature Shows Biomarker Utility in Multiple Myeloma. Int. J. Mol. Sci. *22*, 13144. https://doi.org/10.3390/ijms222313144.
34. Kubiczkova, L., Kryukov, F., Slaby, O., Dementyeva, E., Jarkovsky, J., Nekvindova, J., Radova, L., Greslikova, H., Kuglik, P., Vetesnikova, E., et al. (2014). Circulating serum microRNAs as novel diagnostic and prognostic biomarkers for multiple myeloma and monoclonal gammopathy of undetermined significance. Haematologica *99*, 511–518. https://doi.org/10.3324/haematol.2013.093500.
35. Drees, E.E.E., and Pegtel, D.M. (2020). Circulating miRNAs as Biomarkers in Aggressive B Cell Lymphomas. Trends Cancer *6*, 910–923. https://doi.org/10.1016/j.trecan.2020.06.003.
36. Casanova-Salas, I., Aguilar, D., Cordoba-Terreros, S., Agundez, L., Brandariz, J., Herranz, N., Mas, A., Gonzalez, M., Morales-Barrera, R., Sierra, A., et al. (2024). Circulating tumor extracellular vesicles to monitor metastatic prostate cancer genomics and transcriptomic evolution. Cancer Cell *42*, 1301–1312.e7. https://doi.org/10.1016/j.ccell.2024.06.003.
37. Driedonks, T., Jiang, L., Carlson, B., Han, Z., Liu, G., Queen, S.E., Shirk, E. N., Gololobova, O., Liao, Z., Nyberg, L.H., et al. (2022). Pharmacokinetics and biodistribution of extracellular vesicles administered intravenously and intranasally to Macaca nemestrina. J. Extracell. Biol. *1*, e59. https://doi.org/10.1002/jex2.59.
38. Ye, T., Mishra, A.K., Banday, S., Li, R., Hu, K., Coleman, M.M., Shan, Y., Chowdhury, S.R., Zhou, L., Pak, M.L., et al. (2024). Identification of WNK1 as a therapeutic target to suppress IgH/MYC expression in multiple myeloma. Cell Rep. *43*, 114211. https://doi.org/10.1016/j.celrep.2024.114211.
39. Davis, L.N., Walker, Z.J., Reiman, L.T., Parzych, S.E., Stevens, B.M., Jordan, C.T., Forsberg, P.A., and Sherbenou, D.W. (2024). MYC Inhibition Potentiates CD8+ T Cells Against Multiple Myeloma and Overcomes Immunomodulatory Drug Resistance. Clin. Cancer Res. *30*, 3023–3035. https://doi.org/10.1158/1078-0432.CCR-24-0256.
40. Tang, B., Xu, A., Xu, J., Huang, H., Chen, L., Su, Y., Zhang, L., Li, J., Fan, F., Deng, J., et al. (2018). MicroRNA-324-5p regulates stemness, pathogenesis and sensitivity to bortezomib in multiple myeloma cells by targeting hedgehog signaling. Int. J. Cancer *142*, 109–120. https://doi.org/10.1002/ijc.31041.
41. Bianchi, G. (2022). Sustained minimal residual disease in myeloma. Blood *139*, 469–471. https://doi.org/10.1182/blood.2021013199.
42. Gonzalez-Martin, A., Adams, B.D., Lai, M., Shepherd, J., Salvador-Bernaldez, M., Salvador, J.M., Lu, J., Nemazee, D., and Xiao, C. (2016). The microRNA miR-148a functions as a critical regulator of B cell tolerance and autoimmunity. Nat. Immunol. *17*, 433–440. https://doi.org/10.1038/ni.3385.
43. Pracht, K., Meinzinger, J., Schulz, S.R., Daum, P., Côrte-Real, J., Hauke, M., Roth, E., Kindermann, D., Mielenz, D., Schuh, W., et al. (2021). miR-148a controls metabolic programming and survival of mature CD19-negative plasma cells in mice. Eur. J. Immunol. *51*, 1089–1109. https://doi.org/10.1002/eji.202048993.
44. Kumar, S., Paiva, B., Anderson, K.C., Durie, B., Landgren, O., Moreau, P., Munshi, N., Lonial, S., Bladé, J., Mateos, M.V., et al. (2016). International Myeloma Working Group consensus criteria for response and minimal residual disease assessment in multiple myeloma. Lancet Oncol. *17*, e328–e346. https://doi.org/10.1016/S1470-2045(16)30206-6.
45. Aparicio-Puerta, E., Gómez-Martín, C., Giannoukakos, S., Medina, J.M., Scheepbouwer, C., García-Moreno, A., Carmona-Saez, P., Fromm, B., Pegtel, M., Keller, A., et al. (2022). sRNAbench and sRNAtoolbox 2022 update: accurate miRNA and sncRNA profiling for model and non-model organisms. Nucleic Acids Res. *50*, W710–W717. https://doi.org/10.1093/nar/gkac363.
46. Fromm, B., Høye, E., Domanska, D., Zhong, X., Aparicio-Puerta, E., Ovchinnikov, V., Umu, S.U., Chabot, P.J., Kang, W., Aslanzadeh, M., et al. (2022). MirGeneDB 2.1: toward a complete sampling of all major animal phyla. Nucleic Acids Res. *50*, D204–D210. https://doi.org/10.1093/nar/gkab1101.
47. Aparicio-Puerta, E., Gómez-Martín, C., Giannoukakos, S., Medina, J.M., Marchal, J.A., and Hackenberg, M. (2020). mirnaQC: a webserver for comparative quality control of miRNA-seq data. Nucleic Acids Res. *48*, W262–W267. https://doi.org/10.1093/nar/gkaa452.
48. Zhang, Y., Parmigiani, G., and Johnson, W.E. (2020). ComBat-seq: batch effect adjustment for RNA-seq count data. NAR Genom. Bioinform. *2*, lqaa078. https://doi.org/10.1093/nargab/lqaa078.
49. Martin, M. (2011). Cutadapt removes adapter sequences from high-throughput sequencing reads. EMBnet. j. *17*, 10–12. https://doi.org/10.14806/ej.17.1.200.
50. Andrews, S. FastQC: A Quality Control Tool for High Throughput Sequence Data. https://www.bioinformatics.babraham.ac.uk/projects/fastqc/
51. Smith, T., Heger, A., and Sudbery, I. (2017). UMI-tools: Modelling sequencing errors in Unique Molecular Identifiers to improve quantification accuracy. Genome Res. *27*, 491–499. https://doi.org/10.1101/gr.209601.116.
52. Dobin, A., Davis, C.A., Schlesinger, F., Drenkow, J., Zaleski, C., Jha, S., Batut, P., Chaisson, M., and Gingeras, T.R. (2013). STAR: ultrafast universal RNA-seq aligner. Bioinformatics *29*, 15–21. https://doi.org/10.1093/bioinformatics/bts635.
53. Massaro, C., Sensoy, H.N., Mulders, M., De Schrijver, C., Gómez-Martín, C., Simon-Nieto, J., Lagerweij, T., Atmopawiro, A., Pérez-Boza, J., Bebelman, M., et al. (2024). Tumor-secreted extracellular vesicles counteract therapy response by triggering inflammatory mesenchymal stem cell development. Clin. Cancer Res. *30*, 4714–4728. https://doi.org/10.1158/1078-0432.CCR-23-4097.
54. Yang, A., Bofill-De Ros, X., Shao, T.J., Jiang, M., Li, K., Villanueva, P., Dai, L., and Gu, S. (2019). 3′ Uridylation Confers miRNAs with Non-canonical Target Repertoires. Mol. Cell *75*, 511–522.e4. https://doi.org/10.1016/j.molcel.2019.05.014.
55. Friedman, J., Hastie, T., and Tibshirani, R. (2010). Regularization Paths for Generalized Linear Models via Coordinate Descent. J. Stat. Softw. 33, 1–22.
56. Robin, X., Turck, N., Hainard, A., Tiberti, N., Lisacek, F., Sanchez, J.C., and Müller, M. (2011). pROC: An open-source package for R and S+ to analyze and compare ROC curves. BMC Bioinf. *12*, 77. https://doi.org/10.1186/1471-2105-12-77.
57. Kern, F., Aparicio-Puerta, E., Li, Y., Fehlmann, T., Kehl, T., Wagner, V., Ray, K., Ludwig, N., Lenhof, H.-P., Meese, E., and Keller, A. (2021). miRTargetLink 2.0—interactive miRNA target gene and target pathway networks. Nucleic Acids Res. *49*, W409–W416. https://doi.org/10.1093/nar/gkab297.
58. Newman, A.M., Steen, C.B., Liu, C.L., Gentles, A.J., Chaudhuri, A.A., Scherer, F., Khodadoust, M.S., Esfahani, M.S., Luca, B.A., Steiner, D., et al. (2019). Determining cell type abundance and expression from bulk tissues with digital cytometry. Nat. Biotechnol. *37*, 773–782. https://doi.org/10.1038/s41587-019-0114-2.

STAR★METHODS

KEY RESOURCES TABLE

REAGENT or RESOURCE	SOURCE	IDENTIFIER
Antibodies		
H5C6 anti human CD63	BD biosciences	Cat#556019; Mouse; RRID:AB_396297
JS-81 anti human CD81	BD biosciences	Cat#555675; Mouse; RRID:AB_396028
D2V7J anti human Flotillin 1	Cell Signaling	Cat#18634; Rabbit; RRID:AB_2773040
Anti human Syntenin	Abcam	Cat#ab19903; Rabbit; RRID:AB_445200
Anti human Calnexin	Merck	Cat#AB2301; Rabbit; RRID:AB_10948000
HRP-conjugated anti-rabbit IgG	Cell Signaling	Cat#7074S; Goat; RRID:AB_2099233
Anti-mouse IgG	DAKO	Cat#P0260; Rabbit; RRID:AB_2636929
Deposited data		
miRNA-seq Multiple Myeloma patients and Healthy donors sequenced sequenced with IsoSeek protocol, data generated for this paper	SRA	PRJNA1183899
Total RNA-seq Multiple Myeloma patients and healthy donors, data generated for this paper	SRA	PRJNA1183899
Software and algorithms		
Models described in the manuscript	Zenodo	https://doi.org/10.5281/zenodo.15319148
Bulk RNAseq deconvolution	CibersortX	https://doi.org/10.1038/s41587-019-0114-2
Particle measurement	Izon Control Suite software	Version 1.0.2.32

EXPERIMENTAL MODEL AND STUDY PARTICIPANT DETAILS

Clinical plasma samples processing and legislation

Blood samples for optimizing the library preparation protocol were collected in EDTA plasma collection tubes (BD Vacutainer) and processed within two hours of collection. Platelet-free plasma was isolated by sequential centrifugation for 7 min at 900g and 10 min at 2500g at room temperature. Plasma was stored in 1 mL aliquots at −80°C until further use. Freeze-thaw cycles were avoided. Samples were collected through biobanking.

Blood from patients with MM was collected following the same procedure. EDTA plasma was stored in 1 mL aliquots at −80°C until further use, and freeze-thaw cycles were avoided. RRMM samples were obtained from patients who participated in the NIVO-DARA trial, DARA-ATRA trials, or from prospective and retrospectively collected biobank samples, all of which were approved by the ethics committees of the participating institutions and were conducted in accordance with the Declaration of Helsinki and Good Clinical Practice guidelines. All patients provided written informed consent for the collection and use of their samples for research purposes.

In the case of the two AMC B cell malignancies biobank samples, plasma isolation was performed using the following protocol: Platelet-free plasma was isolated by sequential centrifugation for 10 min at 2500 rpm and 10 min at 13200 rpm at room temperature. Plasma was stored in 2 mL aliquots at −80°C until further use. Freeze-thaw cycles were avoided. Samples were processed within four hours of blood-draw.

Age- and gender-matched healthy donor samples were collected from Stibion biobank at two different locations, and processed within 4 h of blood-draw following the same protocol.

A summary of all samples included in the study can be found in Table 1.

METHOD DETAILS

Definitions of the clinical sample groups

Samples were categorized by the presence or absence of active MM. Active disease was defined as either untreated, newly diagnosed MM, or the development of PD or refractory disease following the initiation of treatment. Response to treatment was defined as either PR, very good partial response, or complete response, according to the IMWG criteria.[44]

For the pre-treatment response prediction model (Figure 5), samples collected at the start of treatment were analyzed, with responses evaluated at the 6-month mark. Patients who had a PR or better at 6 months were included in the durable response group, while the remaining patients were classified as non-responder i.e., early relapsed/refractory.

Plasma extracellular vesicle isolation

Plasma extracellular vesicles (pEVs) from MM patients and healthy donors were isolated via an automated SEC procedure (AFC Izon Science Limited), using a qEVoriginal 70 nm column. Briefly, 0.5 mL of PBS was added to 1 mL of plasma. The total loaded volume on the column was 1.5 mL. On a qEV original 70 nm column (SP1-EUR, IZON), using a buffer volume of 2.85 mL, the particles of interest were collected in fractions 3 and 4 both of 0.5 mL (between 1 and 2 mL of the Purified Collection Volume). Details on this quality-controlled EV isolation procedure were described previously.[13]

Western blot

Plasma EVs were concentrated using Amicon Ultra 2 mL 10K centrifugal filters (UFC201024; Merck), run on a 4–15% Mini-PROTEAN TGX gel (4561084; Bio-Rad) and blotted on a nitrocellulose membrane. For the detection of CD63 and CD81 SDS-PAGE was performed under non-reducing conditions. Membranes were probed with antibodies against CD63 (mouse; H5C6; 556019; BD), CD81 (mouse; JS-81; 555675; BD), Flotillin 1 (rabbit; D2V7J; 18634; Cell Signaling), Syntenin (rabbit; ab19903, Abcam) and Calnexin (rabbit; AB2301; Merck), followed by secondary antibodies HRP-conjugated anti-Rabbit IgG (goat; 7074S; Cell Signaling) and anti-Mouse IgG (rabbit; P0260; DAKO). Protein expression was visualized using ECL substrate (32209; Pierce) and a ChemiDoc MP Imaging System (Bio-Rad).

Transmission Electron microscopy

EV fractions were spotted on freshly glow-discharged carbon/formvar-coated mesh grids. After blotting off the excess liquid, the samples were contrasted by 2% uranyl acetate (Polysciences Inc, Cat No 21447-25) in water for 1 min. The excess stain was blotted off and grids were airdried. Vesicular structures were imaged in a 60 kV JEOL1010 (JEOL) Transmission Electron microscopy (TEM) at 60000x magnification using a 4k x 2.6k pixel CCD side-mounted camera (Modera, EMsis GmbH).

Particle measurement

To determine the EV size distribution and concentration, samples were measured on an Exoid (Izon Science Limited) using a NP150 nanopore. Samples were diluted in electrolyte buffer and measured at 3 different pressures. Concentration and particle size were determined using calibration beads of a known size, and concentrations were measured at the identical settings as the samples. Data were analyzed using the Izon Control Suite software (version 1.0.2.32).

RNA isolation and quality control

RNA from pEVs was isolated using the miRNeasy serum/plasma kit (QIAgen) according to the manufacturer's protocol. Briefly, RNA from bulk plasma EV fractions was isolated using 1 miRNeasy spin column and RNA was eluted in 14 μL nuclease-free water. To determine the quality of the EV-RNA we determined the presence of several amplifiable miRNAs with a QC-qPCR as described previously.[19] Total RNA from cell lines was isolated using TRIzol reagent (Thermo Fisher Scientific) according to the manufacturers' protocol.

Small RNA library preparation and sequencing

IsoSeek was used to prepare small RNA libraries from pEVs as previously described.[19] Briefly, 4 μL of pEVs RNA was used as input and 5N-adapters and RT-primers were diluted 1:50 (5′-5N-adapter 225 nM, 3′-5N-adapter 100 nM, RT-primer 1:50). For the optimization of the workflow, libraries were prepared using the commercial NEBNext Multiplex Small RNA Library Prep Kit for Illumina. The adapters and RT-primer were diluted 1:10 (5′-adapter 1.13 μM, 3′-adapter 0.5 μM, RT-primer 1:10). Libraries were quantified using a KAPA PCR Quantification Kit (Roche, Cat. no 07960298001) and libraries were pooled in equimolar amounts (1.5–2 nM). Sequencing was performed on a NovaSeq6000 platform, PE50 (Healthy donor and patients with MM samples) or SE50 on a HiSeq4000 platform for the optimization samples.

For technical validation samples were re-sequenced on a MiSeq platform, PE50 (1.5 nM).

Total RNA library preparation and sequencing

Total RNA libraries were prepared using the SMARTer Stranded Total RNA-Seq Kit v3 - Pico Input Mammalian (Takara Bio Inc.) according to the manufacturers' protocol, including ribosomal cDNA depletion. 8 μL of pEVs RNA was used as input, followed by a mild fragmentation step of 3 min at 94°C. For the first PCR, samples were subjected to 5 rounds of amplification, the final RNA-seq library amplification PCR consisted of 16 PCR cycles. Sequencing was performed on a NovaSeq6000 platform, PE150.

QUANTIFICATION AND STATISTICAL ANALYSIS

Processing of sequencing data and microRNA profiling

Pre-processing, mapping of adapter trimmed reads and isomiR classification were performed using the latest version of sRNAbench command line tool.[45] Default parameters were used for all analysis steps after pre-processing and MirGeneDB 2.1[46] was used as miRNA reference. Quality control of samples was carried out using mirnaQC[47] to rule out technical differences between libraries. RPM normalized against the miRNA library were used as input for the subsequent analysis (RPMlib). IsomiR classification was performed as previously described.[17,45] Outlier analysis was computed by means of PCA (Figure S1C, outliers highlighted in red circle).

Four healthy control samples were excluded based on this analysis. Batch effect correction was performed in the validation sets using ComBat-seq R package.[48]

Processing and analysis of total RNA sequencing data

Total RNA sequencing data analysis was carried out using Cutadapt v4.5[49] for adapter trimming, FastQC[50] for quality control, Umi tools v1.1.2[51] to perform UMI correction of the reads and STAR[52] to align to the reference transcriptome (GRCh38.p14) as previously described.[53] NormSeq[21] was used to normalize the raw counts.

IsoSeek performance evaluation on plasma extracellular vesicle samples

To assess the robustness of IsoSeek in small RNA profiling in plasma EV samples, we compared its performance with the commercial NEBNext protocol. IsoSeek detected a greater number of miRNAs across all abundance ranges (Figure S2A) and 15,000 isomiRs in each plasma EV sample, with 1,000 isomiRs at least 10 read counts per million (RPM). This is significantly higher than what we obtained with NEBNext (3,000 isomiRs per sample and <500 surpassing the 10 RPM threshold, see Figure S2B).

Detecting miRNAs from low input samples with small RNAseq requires sufficient amplification rounds to achieve an optimal library yield increasing a risk for introducing amplification bias.[19] We applied IsoSeek that corrects for amplification- and adapter-ligation bias enhancing detection accuracy of synthetic miRNAs and isomiRs.[17,19] While comparison of pEVs miRNA detection with and without UMI correction revealed a small effect on classic miRNA detection (Figure S2C), UMI correction has to the profound effect on isomiR detection in pEVs (Figure S2D). Additionally, differences in normalized miRNA reads between technical replicates of a single pEV sample were reduced after UMI correction (Figure S2H). Moreover, IsoSeek provides a gradual increase in accumulative reads, particularly for isomiRs (Figure S2E), indicating improved accuracy in capturing actual miRNA/isomiR sequence distribution in plasma EVs samples. Therefore, for clinical samples with low RNA input, such as plasma EVs, IsoSeek may mitigate biased detection and quantification of isomiRs.

Analysis of NTA subclasses revealed that IsoSeek detected increased levels of NTA-U and only very low levels of NTA-C as compared to NEBNext (Figure S2I), consistent with previous findings.[17] This aligns with NTA-C representing an enigmatic isomiR class with unknown biological relevance. We thus focused on the level of uridylation (NTA-U) and adenylation (NTA-A) for each individual miRNA, as these modifications are known to deviate from the canonical targetome of the mature miRNA sequence.[54] Significant differences between IsoSeek and the conventional protocol were observed when taking the modifications into account (Figures S2F and S2G).

Statistical modeling

Each classifier model was built using the miRNA RPMlib matrix (normalized against the miRNA library size per sample) of all the samples as input data. Two different possible input miRNAs matrices were used, classic miRNA annotation (not taking into account isomiR information), and canonical miRNAs + NTA-U/A isomiRs (isomiR annotation). In each case, a cut-off of 10 RPM per miRNA in at least the number of samples of the smaller group was applied. The R-package *glmnet* was used to compute the lasso (logistic regression and penalization/shrinkage of the parameters) penalized regression models.[55] In each model, data were split into train (2/3 of the samples) and validation (1/3 of the samples) sets. Each model was built in the training set using a 6-fold cross-validated approach (*cv.glmnet*). Finally, each model was tested in the validation data and also in the external validation cohorts and the test ROCs of the different models were computed using the *pROC* R-package.[56] CI-Interval was calculated with 1000-fold bootstrapping.

MicroRNA target network analysis

MiRNA target network analysis was performed using miRTargetLink 2.0[57] using the miRNAs in each different model as input. Targetome of NTA-U isomiRs (conserved TUMR targets) was obtained as previously described.[54] Network visualization was performed with Cytoscape v3.10.2.

Survival analysis

PFS and OS were plotted using the Kaplan-Meier method. Survival analyses were conducted using the Cox proportional hazards regression analysis.

Deconvolution analysis

Deconvolution analysis was done using CIBERSORTx[58] software using LM22 single-cell sequencing reference, and default parameters.

ADDITIONAL RESOURCES

The Biolymph study was registered in the Dutch CCMO-register as NL60245.029.17: https://toetsingonline.nl.

The DARA-ATRA and NIVO-DARA studies were registered as NCT02751255 and NCT03184194, respectively: https://www.ClinicalTrials.gov.

The AMC B cell malignancies biobank samples are registered with the local Medical Ethics testing committee under number METC 2013_159.

Cell Reports Medicine, Volume *6*

Supplemental information

Circulating extracellular vesicle isomiR signatures predict therapy response in patients with multiple myeloma

Cristina Gómez-Martín, Esther E.E. Drees, Monique A.J. van Eijndhoven, Nils J. Groenewegen, Steven Wang, Sandra A.W.M. Verkuijlen, Jan R.T. van Weering, Ernesto Aparicio-Puerta, Leontien Bosch, Kris A. Frerichs, Christie P.M. Verkleij, Marie J. Kersten, Josée M. Zijlstra, Daphne de Jong, Catharina G.M. Groothuis-Oudshoorn, Michael Hackenberg, Johan R. de Rooij, Niels W.C.J. van de Donk, and D. Michiel Pegtel

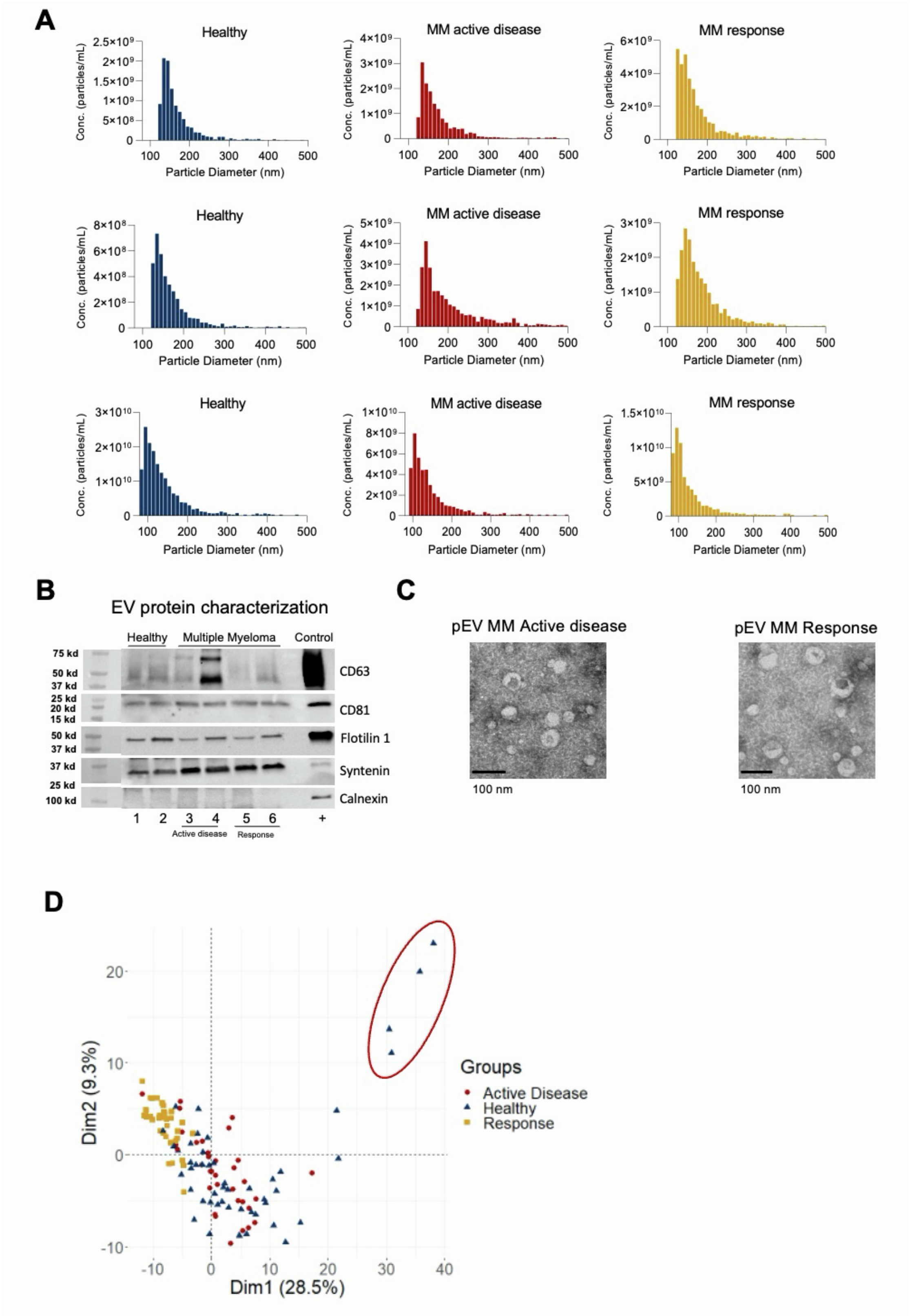

Figure S1. EV characterization. Related to Figure 1 and STAR methods. A) Particle size distribution of plasma EVs from healthy donors (left panels), MM patients with active disease (middle panels), and MM patients who achieved response to therapy (right panels), using Exoid. **B)** Western blot analysis of plasma EVs from healthy donors and MM patients with active disease or with clinical response. A B-cell lysate serves as a positive control. **C)** TEM analysis of pEVs from patients with MM active disease (left) or clinical response (right). Scale bar indicates 100 nm. **D)** PCA of all included samples, showing 4 outliers (red circle) belonging to healthy group.

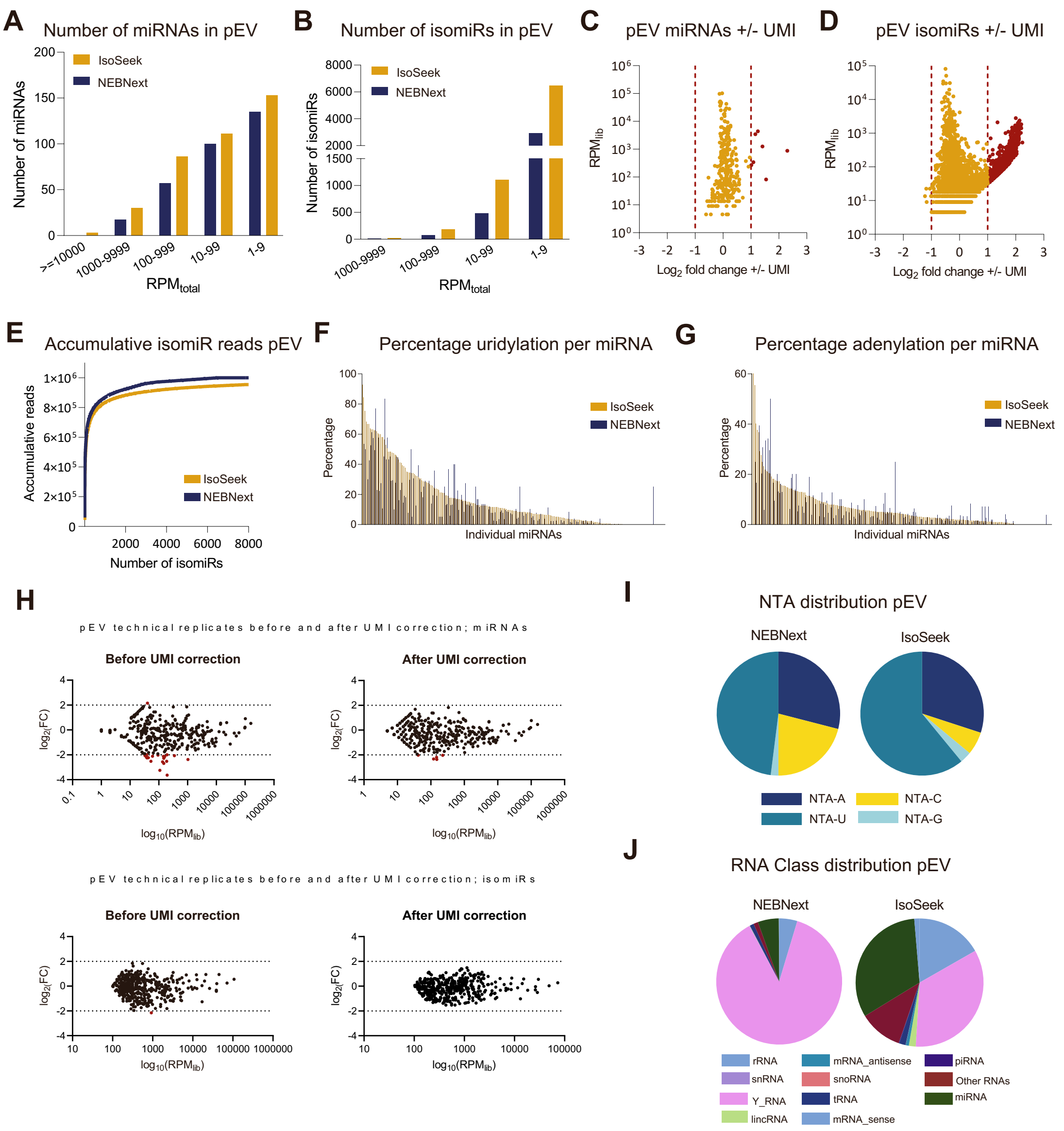

Figure S2. IsoSeek improves the detection of miRNA and variants (isomiRs) at single nucleotide resolution in patient plasma EVs. Related to Figure 1 and STAR methods. A) Number of different miRNAs detected in pEVs libraries prepared with NEBNext (blue) or IsoSeek (yellow), sorted by abundance (RPM_{total}). Data shown is the average of n=2 for each procedure. **B)** Same as in (A) but for isomiRs as separate features. **C)** Differential expression analysis of pEVs-miRNAs using 5N-adapters with or without UMI correction. Each dot represents a miRNA. Representative data is shown. **D)** Same as in (C) but for isomiRs as separate features. **E)** Accumulative normalized isomiR reads from pEVs libraries prepared using NEBNext (blue) and IsoSeek (yellow). The results shown are the average of n=2 for both procedures. **F)** Percentage of uridylation for each miRNA in pEVs libraries prepared using NEBNext (blue) or IsoSeek (yellow). The NTA-U reads were divided by the total normalized reads for each miRNA. Each line represents a miRNA, sorted by abundance based on IsoSeek. Data shown is the average of n=2 for each library preparation procedure. Analysis includes miRNAs >= 10 RPM (total reads) in all samples. **G)** Same as in (F) but for the percentage of adenylation. **H)** Differential expression analysis of pEVs-miRNAs (top) or pEVs-isomiRs (bottom) of technical replicates using 5N-adapters with UMI (blue) or without UMI correction (red). Each line represents a miRNA, sorted by abundance based on the DEA with UMI correction. Representative data is shown. Analysis only includes miRNAs detected in both samples, with a cut-off for miRNAs (top) of 1RPM and a cut-off for isomiRs (bottom) of 100 RPM. **I)** NTA distribution in pEVs libraries prepared with NEBNext (left) and IsoSeek (right). Data shown is the average of n=2 for each procedure. **J)** Small RNA species distribution in pEVs libraries prepared with NEBNext (left) and IsoSeek (right). Data shown is the average of n=2 for each procedure.

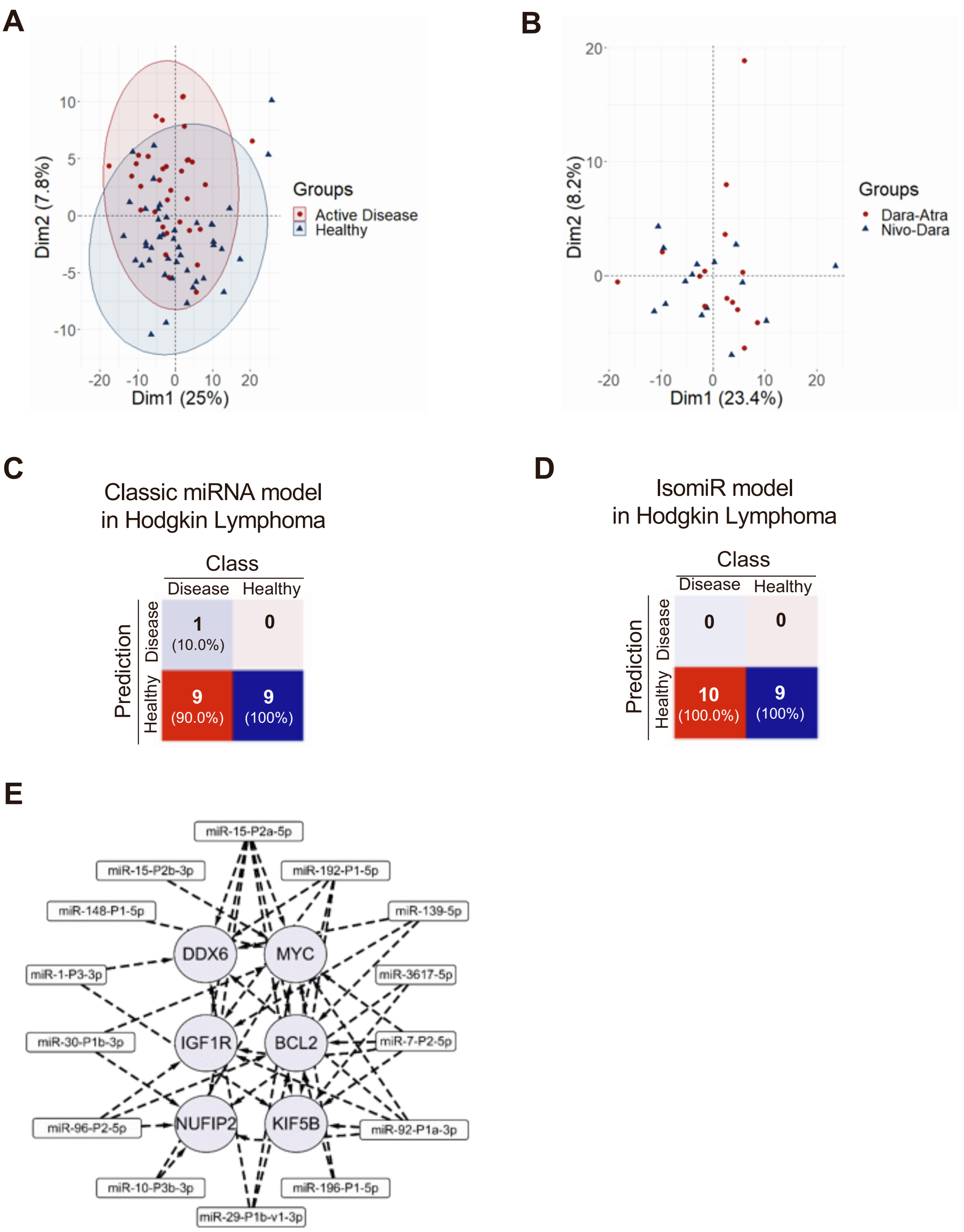

Figure S3. Related to Figure 2. A) Principal component analysis with all samples from MM patients with active disease and from healthy donors. **B)** Principal component analysis with all samples from MM patients with active disease annotated by treatment **C** and **D)** Performance of the classic miRNA MM disease model **(C)** and isomiR model **(D)** on Hodgkin Lymphoma active disease samples as well as a set of healthy controls. **E)** Full miRNA-gene-target network of the classic miRNA disease detection model.

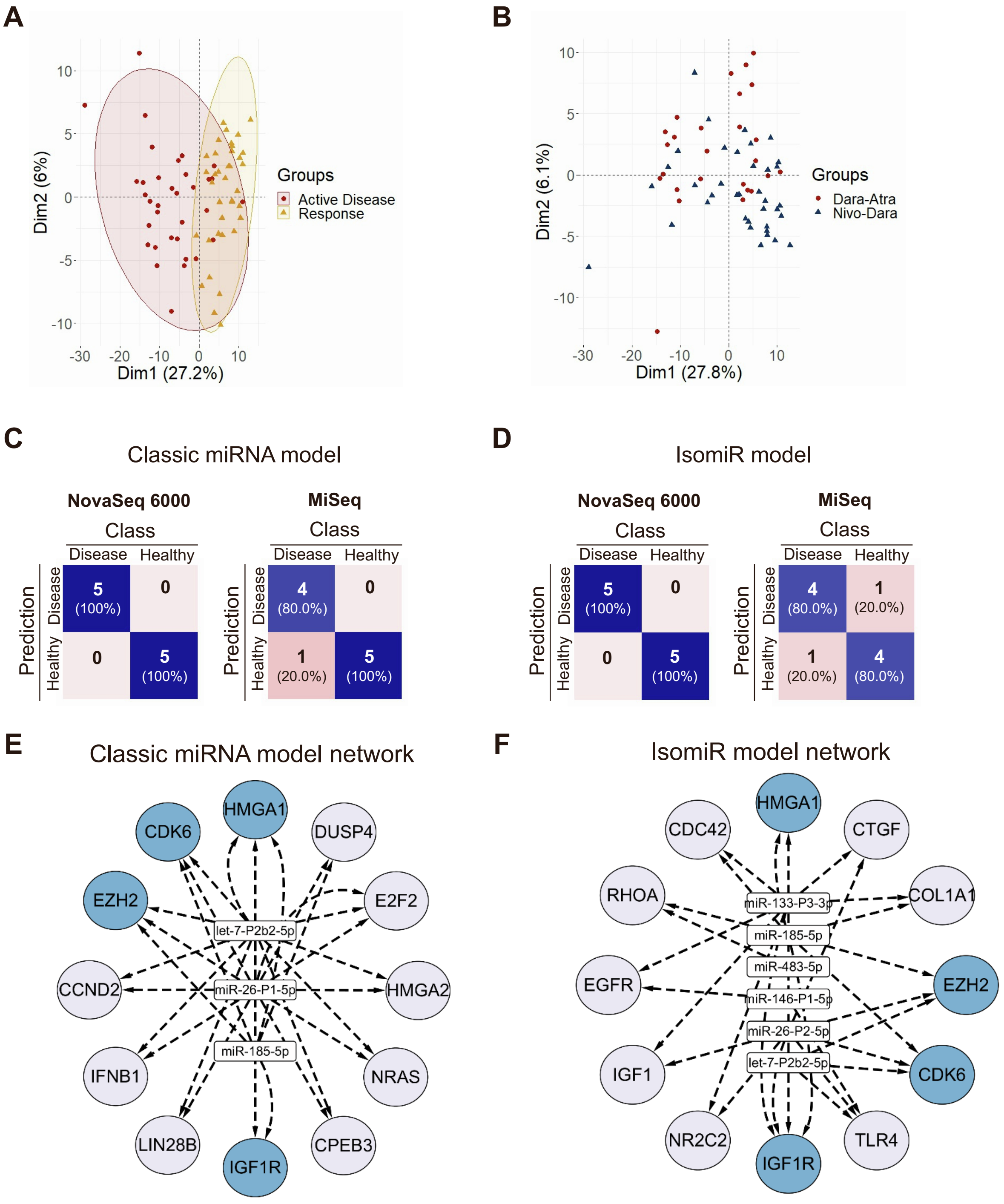

Figure S4. Related to Figure 3. A) Principal component analysis with all samples from MM patients with active disease and with clinical response. **B)** Principal component analysis with all MM samples (AD and response) annotated by treatment. **C-D)** Performance of the classic miRNA MM disease model **(C)** and isomiR model **(D)** on resequenced samples using the MiSeq sequencing platform. **E)** Full miRNA-gene-target network of the classic miRNA disease detection model. **F)** Full miRNA-gene-target network of the IsomiR miRNA disease detection model.

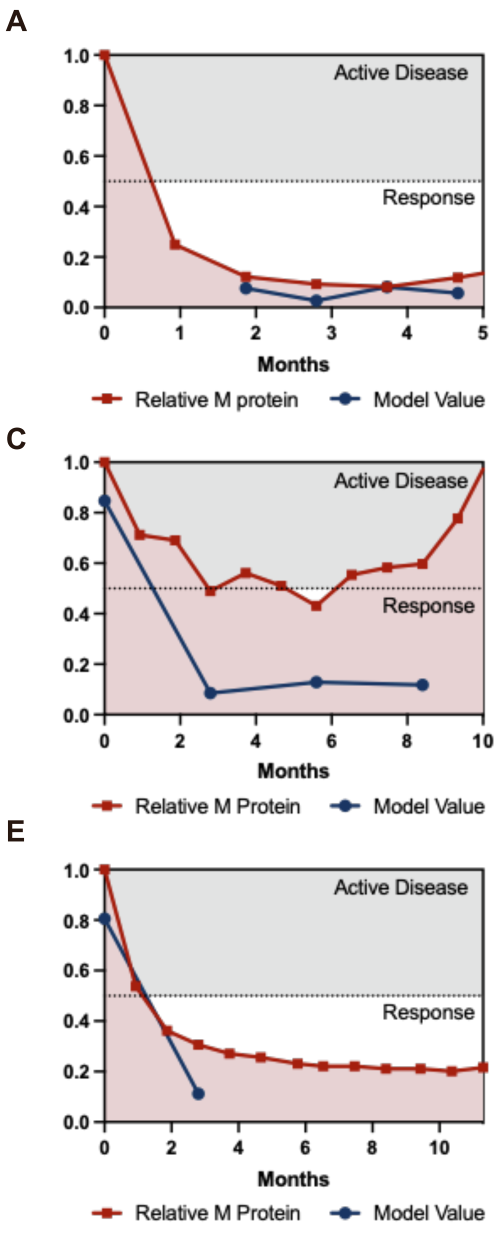

Figure S5. Related to Figure 4. A-E) pEV-isomiR model predictions over time compared to M-protein levels for four individual patients, showing that the model closely tracks the M-protein metric, demonstrating its robustness.

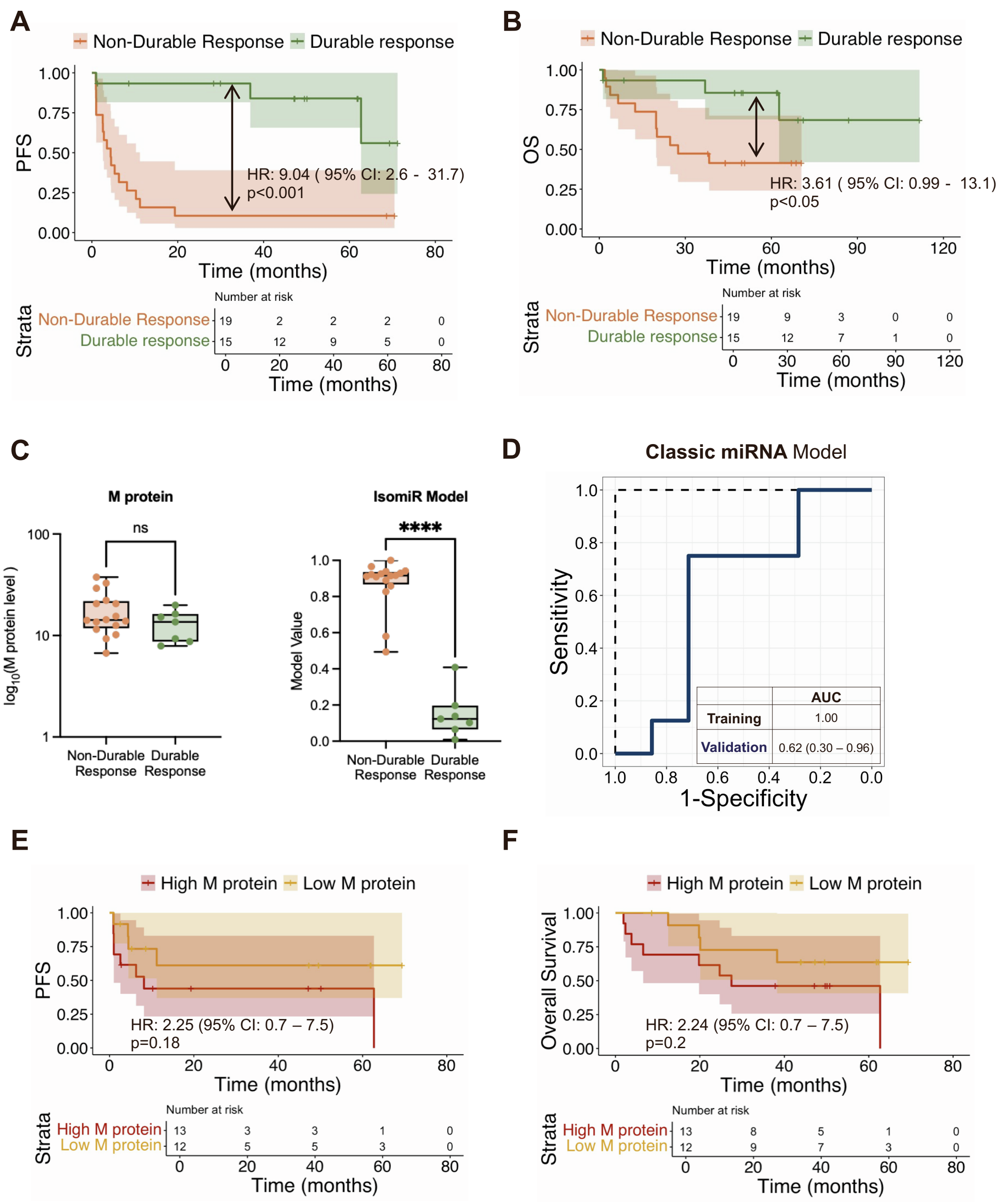

Figure S6. Related to Figure 5. A) Progression-free survival (PFS) of the total set of patients included in the model in Figure 6, including both RRMM and NDMM patients (n=34). The survival curve was computed using the Kaplan-Meier method. The hazard ratio (HR) calculated by Cox-regression is 9.04 (CI:2.6-31.7, p<0.001). **B)** Overall survival (OS) analysis of the total set of patients included in the model in Figure 6, including both RRMM and NDMM patients (n=34). The survival curve was computed using the Kaplan-Meier method. The hazard ratio (HR) calculated by Cox-regression is 3.61 (CI:0.99-13.1, p<0.05). **C)** (Left) Log10-transformed baseline M-protein levels in Non-Durable Response (orange) and Durable Response (green) patients. No significant difference was observed (t-test, n.s.) between the two groups. (Right) IsomiR prediction model value in Non-Durable response (orange) and Durable Response (green). Significant difference was observed (t-test, p-value < 0,0001) between the two groups. **D)** ROC curve of the classic miRNA durable response prediction model. The training model (dotted line) had and AUC of 1.0, but could not be validated in the independent validation set (AUC: 0.62). **E)** Progression-free survival (PFS) of RRMM patients (n=25) divided by M-protein values (high or low). The survival curve was computed using the Kaplan-Meier method. The hazard ratio (HR) calculated by Cox-regression is 2.25 (CI:0.7-7.5, p=0.18). **F)** Overall survival (OS) analysis of RRMM patients (n=25). The survival curve was computed using the Kaplan-Meier method. The hazard ratio (HR) calculated by Cox-regression is 2.24 (CI:0.7-7.5, p=0.2).

**Patients with MM included in disease detection model (Figure 2)**	**Training cohort**	**Validation Cohort**
**Number of Samples**	***22 patients<br>22 samples***	***16 patients<br>18 samples***
**Gender**	9 male<br>13 female	11 male<br>5 female
**Age**	Median: 62 yrs<br>Range: 47-80 yrs	Median: 66 yrs<br>Range: 54-80 yrs
**Treatment line**	Median: 5<br>Range: 0-11	Median: 4<br>Range: 0-9
**Treatment regimen (treatment at time of collection samples)**		
**RRMM Daratumumab trial patients**		
NIVO-DARA +/- low-dose cyclophosphamide	8	7
DARA-ATRA	10	8
**Biobank (Biolymph VUmc)**		
NDMM		
VTd followed by HDM/ASCT	2	0
VCd followed by HDM/ASCT	1	1
RRMM		
Daratumumab (+/- lenalidomide/dexamethasone)	1	0
**Monoclonal protein**		
IgG kappa	14	11
IgG lambda	3	1
IgA kappa	1	0
IgA lambda, non/oligo-secretor	1	1
FLC kappa	3	2
FLC lambda	0	1
**High-risk cytogenetic abnormality**		
High risk	12	7
Standard risk	4	3
Unknown	6	6

**Healthy donors included in disease detection model (Figure 2)**	**Training cohort**	**Validation Cohort**
**Gender**	14 male<br>15 female	9 male<br>6 female
**Age**	Median: 63 yrs<br>Range: 45 – 78 yrs	Median: 61 yrs<br>Range: 48 – 79 yrs

Table S1: Response status of the training and validation set for disease detection model in Figure 2. **Abbreviations:** HDM = high-dose melphalan; ASCT = autologous stem cell transplantation; NIVO = nivolumab; DARA = daratumumab; ATRA = all trans retinoic acid; VTd = Bortezomib-thalidomide-dexamethasone; VCd = Bortezomib-cyclophosphamide-dexamethasone. Based on the criteria proposed by Sonneveld et al. Blood 2016. High risk cytogenetics is defined by the presence of t(4;14), t(14;16), t(14;20), del(17/17p), and/or gain(1q).

Patients with MM included in response assessment model (Figure 3)	Training set RRMM		Validation RRMM		Prospective validation RRMM and NDMM	
	Active Disease	*Response*	*Active Disease*	*Response*	*Active Disease*	*Response*
	20 patients 20 samples	***11 patients 22 samples***	***13 patients 15 samples***	***7 patients 13 samples***	***6 patient 6 samples***	***11 patients 11 samples***
**Gender**	9 male 11 female	3 male 8 female	8 male 5 female	3 male 4 female	3 male 3 female	7 male 4 female
**Age**	Median: 67 yrs Range: 47-80 yrs	Median: 60 yrs Range: 55-77 yrs	Median: 66 yrs Range: 54-80 yrs	Median: 62 yrs Range: 54-74 yrs	Median: 59 yrs Range: 57-87 yrs	Median: 63 yrs Range: 34-80 yrs
**Treatment line**	Median: 4 Range: 2-11	Median: 5 Range: 3-8	Median: 4 Range: 3-9	Median: 4 Range: 3-8	Median: 0 Range: 0-7	Median: 1 Range: 1-10
**Treatment regimen (treatment at time of sample collection)**						
**RRMM Daratumumab trial patients**						
NIVO-DARA +/- low-dose cyclophosphamide	10	7	5	6		
DARA-ATRA	10	4	8	1		
**Biobank (Biolymph VUmc / AMC)**						
NDMM						
VTd followed by HDM/ASCT					2	0
VCd followed by HDM/ASCT					2	0
Lenalidomide maintenance after HDM/ASCT					0	2
KTd followed by HDM/ASCT (CarthaDex trial)					0	0
KRd followed by HDM/ASCT (HOVON503 trial, arm B)					0	1
RRMM						
Daratumumab (+/-lenalidomide/dexamethasone)					2	2
Standard of care CARTITUDE-4 trial regimen					0	1
TRIMM-2 trial					0	1
VCd followed by DLI					0	1
PCd					0	1
EPd					0	1
DVd					0	1
**Monoclonal protein**						
IgG kappa	13	7	9	4	5	3
IgG lambda	4	2	0	1	0	0

IgA kappa	1	0	0	0	0	1
IgA lambda, non/oligo-secretor	0	0	1	0	0	2
FLC kappa	1	2	3	2	1	5
FLC lambda	1	0	0	0	0	0
**High-risk cytogenetic abnormality**						
High risk	12	6	7	4	0	2
Standard risk	2	2	3	1	2	1
Unknown	7	3	3	2	4	8

**Distribution of the samples per analysis group**						
**Clinical trial samples**						
**Active Disease (AD) Group**	**20**		**15**			
NIVO-DARA (ND) baseline	10		6			
DARA-ATRA (DA) baseline	6		8			
DARA-ATRA (DA) post ATRA	4		1			
**Response group**		**22**		**13**		
Partial response		10		7		
Very good partial response		8		4		
Complete response		4		2		
Biobank Samples						
**Active Disease (AD) Group**					**6**	
Newly Diagnosed					4	
Progressive disease					2	
**Response group**						**11**
Stable disease						1
Partial response						2
Very good partial response						3
Complete response						5

Table S2: Response status of the training, validation and prospective validation set for the response assessment model from Figure 3.
Abbreviations: HDM = high-dose melphalan; ASCT = autologous stem cell transplantation; NIVO = nivolumab; DARA = daratumumab; ATRA = all trans retinoic acid; VTd = Bortezomib-thalidomide-dexamethasone; VCd = Bortezomib-cyclophosphamide-dexamethasone; KTd = Carfilzomib-thalidomide-dexamethasone; KRd = Carfilzomib-lenalidomide-dexamethasone ; Standard of care CARTITUDE-4, Arm = either bortezomib or daratumumab, with pomalidomide and dexamethasone; TRIMM-2 trial = Talquetamab– daratumumab–pomalidomide; DLI = donor lymphocyte infusion; PCd = Pomalidomide-cyclophosphamide-dexamethasone, EPd = Elotuzumab-pomalidomide-dexamethasone; DVd = Daratumumab-bortezomib-dexamethasone. High risk cytogenetics is defined by the presence of t(4;14), t(14;16), t(14;20), del(17/17p), and/or gain(1q). Second part of the table depicts the sample distribution between the different cohorts including the response status of the sample at time of sample inclusion in the model.
